# Genome-Wide Identification and Analysis of Chitinase GH18 Gene Family in *Valsa mali*

**DOI:** 10.3390/jof11040290

**Published:** 2025-04-07

**Authors:** Gulnaz Kahar, Yakupjan Haxim, Xuechun Zhang, Xiaojie Liu, Huawei Liu, Xuejing Wen, Xiaoshuang Li, Daoyuan Zhang

**Affiliations:** 1 Xinjiang Key Laboratory of Conservation and Utilization of Plant Gene Resources, Xinjiang Institute of Ecology and Geography, Chinese Academy of Sciences, Urumqi 830011, China; gulinazikahaer20@mails.ucas.ac.cn (G.K.); .lixs@ms.xjb.ac.cn (X.L.); 2 Key Laboratory of Integrated Pest Management on Crops in Northwestern Oasis, Ministry of Agriculture and Rural Affairs, Institute of Plant Protection, Xinjiang Uygur Autonomous Region Academy of Agricultural Sciences/Xinjiang Key Laboratory of Agricultural Biosafety, Urumqi 830091, Xinjiang, China; 3 University of Chinese Academy of Sciences, Beijing 100049, China; 4 Turpan Eremophytes Botanical Garden, Chinese Academy of Sciences, Turpan 838008, China; 5 State Key Laboratory of Desert and Oasis Ecology, Xinjiang Institute of Geography and Ecology, Chinese Academy of Sciences, Urumqi 830011, China; 6 School of Life Sciences, Xinjiang Normal University, Urumqi 830011, China

**Keywords:** chitinase, *Valsa mali*, gene family, canker

## Abstract

Chitinases are enzymes that catalyze the hydrolysis of chitin and play a significant biophysiological role in fungal growth, development, and pathogenesis. *Valsa mali* is a necrotrophic fungus that is a primary contributor to apple *Valsa* canker. Our study focused on the identification of chitinase gene families from *V. mali* and the analysis of their expression profiles during infection and nutritional growth. A phylogenetic analysis and conservation of catalytic domains were used to classify these genes into three classes, and their chromosome distribution was random. The qRT-PCR analysis identified five differentially expressed VmGH18 genes during infection and nutritional growth. GH18 chitinases use glutamate, whereas VmGH18-4 (VM1G_05900) and VmGH18-10 (VM1G_03597) use glutamine as the catalytic motif. To further test whether it can induce cell death in apple, the recombinant protein was produced in *E. coli*. It showed that the purified VmGH18-4 recombinant protein retained cell-death inducing activity, and it could also induce cell death in apple. But the enzyme activity shows that neither VmGH18-4 nor VmGH18-10 have chitinases enzyme activity. These results suggest that VmGH18-4 can elicit cell death in multiple plant species, while VmGH18-10 cannot.

## 1. Introduction

Chitin is a polysaccharide polymer compound produced by the β-linkage polymerization of N-acetylglucosamine monomers [1], and it is widely distributed in the insect exoskeleton and parts of worm eggs, plankton, crustaceans, and mollusk cells [2,3]. Chitin is also a constituent of the cell walls of some fungi [4]. Chitinase (EC 3.2.1.14) is a chitin-specific hydrolase that specifically recognizes and hydrolyzes β-1,4-glycosidic bonds in N-acetylaminoglycans (such as chitin and chitosaccharides), releasing oligomers, dimers (chitosaccharides), and N-acetylglucose monomers [5]. Based on their structures and functional domains, the chitinases can be divided into two major subfamilies, glycoside hydrolase 18 (GH18), and glycoside hydrolase 19 (GH19).

The GH18 family is widely distributed in microorganisms, animals, and plants, while the GH19 family is primarily found in plants and bacteria [6]. Chitinases produced by microorganisms come from a wide range of sources, but most belong to the glycoside hydrolase 18 family and only a few to the glycoside hydrolase 19 family [7]. Despite there being a variety of primary components, all of the GH18 family chitinases contain one catalytic GH18 domain and multiple auxiliary domains, such as chitin-binding domains and a LysM domain. The catalytic domains have a TIM-barrel (β/α) 8-fold, despite possessing distinct basic structures, and they are characterized by a highly conserved signature sequence (DXDXE motif) on the β4-strand [8,9,10]. The GH18 family of chitinase proteins in fungi are divided into three major groups: A, B, and C. Each of these groups can be divided into subgroups: A2–A5, B1–B5, and C1–C2 [11].

There is a close relationship between the structure of the catalytic center and the downstream chitinase-binding domain (CBD) of a chitinase. The CBD and LysM domain in classes B and C are unique compared to those in class A, which only has a catalytic active center [12]. Sabine Gruber et al. found that class C chitinases in mold had a CBM module and LysM module compared with class A chitinases, and the existence of special domains enhanced the ability of the chitinases to bind substrates, thus improving the hydrolysis efficiency of these chitinases [13]. The special conjugation structure of LysM and chitinase activity centers is common in plants and fungi. The mode of LysM’s binding to chitin and the ability to sense the chitin type differ between ferns and plants, which is related to the number of LysM domains and the coordination between binding motifs. Phylogenetic analysis showed that the fern LysM-GH18 monophyletic group appeared earlier than fungi [14]. In addition, some studies have shown that CBM can enhance chitinase synthesis [15].

Fungal chitinase B (ChiB), a GH18 family hydrolase localized in fungal cell walls, plays a significant role in autolytic processes. Comparative studies demonstrate that during the autolytic phase, ChiB-deficient mutants exhibited a slower rate of hyphal biomass degradation (measured by dry weight reduction) compared to wild-type strains [16,17]. Filamentous fungi encode a variety of chitinases with structural and functional differences, and a large proportion of them are related to the recombination and extension of fungal endogenous cell walls [18,19]. The effect of fungal cell wall degradation is obvious, the products formed during decomposition can be used as a carbon source, and chitin is a quality source that is efficiently utilized [20,21]. This process is of great significance to the development, growth, branching, fusion, and self-melting of fungal hyphae [22]. Beyond that, the degradation of the cell wall by filamentous fungal chitinase can also be reflected in the antagonism of fungi against other fungi or in insects and nematodes with chitin as a protective structure [23].

Some studies have also shown that the autolysis process induced by chitinase is closely related to the formation of fungal spores [24]. Emri et al. found that the activity of chitinase was significantly upregulated by FluG signal induction, the cell self-melting phenotype was significantly increased, and the hydrolysate of chitin could provide certain nutrients for spore growth [25,26].

Generally, the number of para-homologous genes in fungi reflects the diversity of fungal chitinase function. Related studies have shown that filamentous ascomycetes have more chitin than yeast-like fungi and usually have 10–30 GH18 genes [11,21,27]. When exploring the functions of five class B GH18 chitinase genes in *Aspergillus fumigatus*, Alcazar-Fuoli et al. found that the chitinase activity of single-gene mutants and multi-gene co-mutants in *Aspergillus fumigatus* was lower than that of the wild-type, but the mutations had no significant impact on the growth and development of the fungus in general. It is implied that such chitinase genes only play a role in biological nutrition, thereby delaying cell death [24].

Moreover, recent studies showed that some fungal GH18 chitinases have lost their enzymatic activity but function as putative pathogenicity factors [28]. Pathogens have developed sophisticated techniques to avoid or interfere with microbial-associated molecular pattern (MAMP)-triggered immunity (MTI) and hence spread infection [29]. Pathogen-/microbe-associated molecular patterns (PAMPs/MAMPs) are recognized by pattern recognition receptors (PRRs) located on the cell surface [30]. Additionally, certain pathogens can secrete cell wall-degrading enzymes that specifically disrupt the structural integrity of plant tissues. Upon tissue damage, residues such as lytic fragments of plant cell walls, extracellular ATP, and nicotinamide adenine dinucleotide (NAD) molecules are released and accumulate on the surface of the plant. These small molecules, which originally composed part of the plant cells or tissues, transform into damage-associated molecular patterns (DAMPs) in the state of injury. Serving as signaling molecules, DAMPs initiate and enhance the recognition mechanism for invading pathogens within the plant. Upon the recognition of MAMPs and DAMPs by PRRs, a series of downstream immune signals are triggered, leading to the activation of pattern-triggered immunity (PTI) [31]. These strategies are mediated by effector molecules [32]. Chitin fragments are strong MAMPs that cause MTI in a variety of plant species [33]. A key component of fungal virulence is the suppression of chitin-triggered immunity [4]. Some ascomycete pathogens use effectors with the chitinase activity domain to hide cell wall fragments that would otherwise be detected by plant receptors, in order to avoid chitin-triggered defenses [28]. In addition, some secreted chitinases of the GH18 family with enzymatic activity are involved in the establishment of the infection by preventing chitin oligomers from acting as elicitors of plant immune responses [34].

*Valsa mali* is a necrotrophic fungal pathogen that can cause canker disease in apple trees and severely affects apple yield in Asian countries such as Japan, Korea, and China, causing economic losses each year [35,36]. *Valsa mali* infects apple trees through small wounds or natural openings on the bark. It secretes various virulence factors such as cell wall-degrading enzymes, toxins, and effectors to overcome the host’s defenses. First, there are cell wall-degrading enzymes such as pectinases and xylanases that break down the plant cell walls, allowing the fungus to penetrate and colonize the tissue. Then, there are toxins that might disrupt the host cells directly. Effectors also seem to play a crucial role. They can target the host immune proteins and suppress the plant’s defense responses. There is also mention of microRNA-like RNAs that regulate both the pathogen’s virulence genes and the host’s immune genes. Signal transduction pathways within the fungus, involving G proteins and mitogen-activated protein kinases, also influence its virulence by regulating the expression of these virulence factors. The disease cycle involves overwintering in diseased tissues and spreading through conidia and ascospores, especially during rainy periods. Overall, the pathogenicity model involves a complex interplay between the fungus’s offensive strategies and the tree’s defense mechanisms, with environmental factors such as humidity playing a significant role in disease progression [37]. The life cycle of *V. mali* includes the following stages: spore stage, *V. mali* survives on diseased residues in the form of conidia or sexual spores, which is conducive to their survival in adverse environments; infection stage, when the conditions (such as temperature and humidity) are suitable, spores germinate and invade the plant through stomata or wounds; growth stage, once infected, the fungus grows in the host, gradually causing tissue necrosis and forming lesions; propagation stage, under suitable environmental conditions, the fungi produce new spores, spread to new plants through wind or rain, and complete the life cycle [38]. Wild forests, especially *Malus sieversii* in China, are also threatened by *V. mali* [39]. Exploration of the pathogenicity and virulence factors of *V. mali* is of great significance for preventing canker disease and protecting endangered wild apple forests.

In this study, the chitinase family members in the genomes of *V. mali* were surveyed and their phylogenetic relationships, gene structure, and gene duplication events were analyzed. In addition, the expression patterns of the chitinase genes in *V. mali* invading *M. sieversii* were analyzed. The present results extend our knowledge of chitinases in filamentous fungal species and might provide effective gene resources for improving apple resistance to the fungal virulence of *V. mali*.

## 2. Materials and Methods

### 2.1. Fungal Growth

The *V. mali* pathogenic strains (03-8) were kindly provided by Prof. Huang LiLi (State Key Laboratory of Crop Stress Biology for Arid Areas, College of Plant Protection, Northwest A&F University), and preserved by the Xinjiang Key Laboratory of Conservation and Utilization of Plant Gene Resources Urumqi, China. The fungal strains were cultured on potato dextrose agar (PDA) medium at 25 °C.

### 2.2. Plant Growth

For *M. sieversii*, we used tissue-cultured plant materials. The existing *M. sieversii* tissue culture plants of our laboratory were selected as experimental materials (cultivated from the explants of the young stem section with axillary buds), and the subculture medium was MS + 0.4 mg/L 6-BA + 0.1 mg/L NAA, as described by Liu et al. [40].

### 2.3. Fungal Infection

Leaf inoculations were performed as described in our previous study [39]. Healthy leaves (3 × 5 mm) were collected from the tissue-cultured plant materials. The leaves were slightly punctured with a fabric pattern wheel (1 cm in diameter) and inoculated with a mycelial plug (5 mm) excised aseptically from the edge of a 5-day-old PDA culture that had been grown at 25 °C. The inoculated leaves were incubated at 25 °C in darkness and under high humidity (90% RH) for 5 days.

### 2.4. Plasmid Construction

The coding sequences of the *VmGH18-2*, *VmGH18-4*, *VmGH18-8,* and *VmGH18-10* genes were amplified by PCR using PrimeSTAR Max DNA Polymerase (TaKaRa, Dalian, China) from the *V. mali* cDNA library. All the sequences were subsequently cloned into pGR107 vectors using an In-Fusion HD Cloning Kit (TaKaRa, Dalian, China). The primers used in this study are listed in Table S1.

### 2.5. Expression and Purification of VmGH18-4 and VmGH18-10

*VmGH18-4* and *VmGH18-10* were amplified and cloned into BamHI and EcoRI sites of the pMAL-c5x vector. *VmGH18-4* and *VmGH18-10* recombinant proteins were expressed in *E. coli* strain BL21 (DE3) cells. Expression was induced by adding 0.25 mM isopropyl-β-D-thiogalactopyranoside (IPTG) for 16 h at 16 °C. Cells were collected by centrifugation at 5000× *g* for 10 min. For protein extraction, cells were resuspended in lysis buffer (20 mM Tris-HCI, 1 mM EDTA, 10 mM maltose monohydrate, pH 7.4) plus 1 mg mL^−1^ lysozyme and 1 mM phenylmethanesulfonyl fluoride (PMSF), followed by sonication and centrifugation at 10,000× *g* for 1 h. VmGH18-4 and VmGH18-10 were purified by affinity chromatography using amylose resin (#E8021; New England Biolabs, Ipswich, MA, USA) following the manufacturer’s instructions. Each protein’s chitinase enzymatic activity was tested using a chitinase activity assay kit (Solarbio, Beijing, China).

### 2.6. Genome-Wide Identification of GH18 Gene Family of V. mali

To identify potential GH18 chitinase genes in *V. mali*, the genome (version JANKOA000000000.1) date was retrieved from the NCBI (https://www.ncbi.nlm.nih.gov/ 26 April 2024). A hidden Markov model seed profile of Glyco_hydro_18 (PF00704) was downloaded from the up-to-date Pfam database [41], and GH18 genes in the genomes were identified using TBtools (II) software (https://github.com/CJ-Chen/TBtools/releases 12 January 2024) [42]. The SMART database was used to confirm the presence of chitinase domains with a cut-off E-value < 0.0001 [43].

### 2.7. Phylogenetic Relationships, Gene Structure, and Conserved Motif Analysis

To study evolutionary relationships, the full-length amino acid sequences of the GH18 proteins from *V. mali* were aligned using Clustal X2.0 (http://www.clustal.org/ 11 January 2023). A phylogenetic tree was generated using MEGA-X software (https://www.megasoftware.net/ 11 May 2023) with the neighbor-joining method, and topological support was assessed through a bootstrap analysis with 1000 replicates. The exon–intron organization of the *VmGH18* genes was visualized using TBtools [42]. Conserved motifs and domains were identified with the MEME Suite [44] and SMART database [43], respectively, and were visualized using TBtools [42]. The domain signatures were identified using the PROSITE database https://prosite.expasy.org/ 13 March 2024 [45]. The SignalP 6.0 online server [46] was used to identify signal peptides in the VmGH18 proteins.

### 2.8. Chromosomal Location Analysis

The location of each *VmGH18* gene on the chromosome and the length of each chromosome were obtained from the NCBI database. The chromosomal locations of the *VmGH18* genes were visualized with TBtools [42] based on genomic annotation data.

### 2.9. RNA Isolation and Real-Time Quantitative PCR Analysis

Total RNA was isolated from each biological sample using a Fungal RNA extract kit (Biomiga, Hangzhou, China). The RNA concentration was measured using a Qubit RNA Assay Kit with a Qubit 2.0 Fluorometer (Life Technologies, Carlsbad, CA, USA). The RNA integrity was assessed using a RNA Nano 6000 Assay Kit with a Bioanalyzer 2100 system (Agilent Technologies, Santa Clara, CA, USA).

The cDNA was synthesized using the TransScript One-Step gDNA Removal and cDNA Synthesis SuperMix (Transgen, Beijing, China). Primers for the GH18 sequences (Table S1) were designed using Primer-BLAST (https://www.ncbi.nlm.nih.gov/tools/primer-blast/ 19 May 2024). *G6PDH* was used as a reference gene for expression [47]. Quantitative reverse transcription PCR was carried out with the PerfectStart^®^ Green qPCR SuperMix (Transgen) on a CFX96 Real-Time PCR Detection System (Bio-Rad, Hercules, CA, USA). The relative gene expression levels were calculated using the 2^−ΔΔCt^ method [48]. Each sample was subject to three biological replicates, and each biological replicate was analyzed with three technical replicates. Statistical analysis of the data was performed with analysis of variance using SPSS 18 software (SPSS, Chicago, IL, USA).

## 3. Results

### 3.1. Genome-Wide Identification and Characterization of VmGH18 Genes in V. mali

To accurately identify the chitinase gene family, functional genome data for *V. mali* were retrieved from the NCBI genome database. The hidden Markov models (HMMs) of the glycoside hydrolase family 18 (PF00704) were downloaded from the Pfam database [49] and scanned through HEMMER3.0 [44]. We identified 17 GH18 genes belonging to the chitinase families in *V. mali* based on the genome. For the 17 VmGH18 members, the whole catalytic domain was obtained.

**Figure 1:**
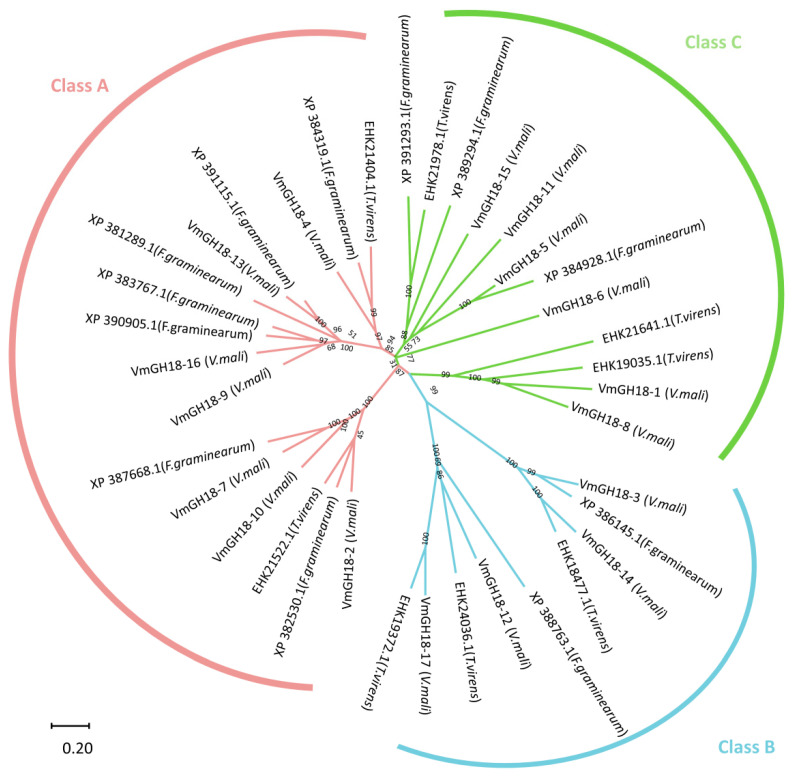
Phylogenetic tree of GH18 genes. Different colors indicate different groups. Red represents group A. Blue represents group B. Green represents group C. The evolutionary history was inferred using the neighbor-joining method. The optimal tree is shown. The percentages of replicate trees in which the associated taxa clustered together in the bootstrap test (1000 replicates) are shown next to the branches. The tree is drawn to scale, with the branch lengths in the same units as those of the evolutionary distances used to infer the phylogenetic tree. The evolutionary distances were computed using the Poisson correction method and are in the units of the number of amino acid substitutions per site. This analysis involved 17 amino acid sequences. All ambiguous positions were removed for each sequence pair (pairwise deletion option). There were a total of 2082 positions in the final dataset. Evolutionary analyses were conducted in MEGA11.

These genes were then designated as *VmGH18-1* to *VmGH18-17* (Table 1). The coding sequence lengths of the 17 VmGH18 genes ranged from 798 (VmGH18-8) to 4710 (*VmGH18-6*) bp. The peptide lengths ranged from 265 (VmGH18-8) to 1569 (*VmGH18-6*) amino acid residues, corresponding to molecular weights (MWs) of 28.94 kDa (*VmGH18-8*) and 170.67 kDa (*VmGH18-6*). The isoelectric points (pIs) ranged from 3.63 (*VmGH18-17*) to 8.39 (*VmGH18-10*). To explore the relationships of the VmGH18 gene family, we generated a phylogenetic tree for the GH18 genes from *Fusarium graminearum* and *Trichoderma virens* as a reference (Table S2). The VmGH18 genes were clustered into three groups, and the A, B and C groups included seven, four, and six genes, respectively (Figure 1).

**Figure 2:**
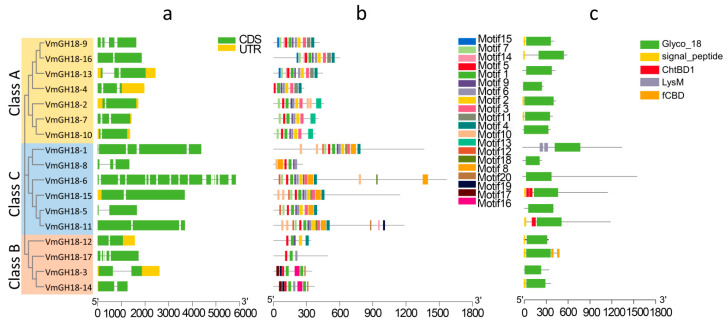
Structure of *VmGH18* genes. (**a**) Gene structure. CDS denotes exons. (**b**) Motif analysis. The lengths and different colors of the boxes denote motif lengths and different motifs, respectively. (**c**) Domain defined using PFAM database. The conserved motifs in GH18 chitinase family proteins from *V. mali* were identified using MEME. Schematic representation of exon–intron structure of GH18 chitinase families in *V. mali*. Active domains of GH18 chitinase proteins from *V. mali* were identified using the SMART database. Motifs, domains and exon–intron structures were visualized using TBtools software. Each single signature is indicated by a colored box at the bottom of the figure and presented proportionally.

In order to better characterize the VmGH18 proteins, the meme was used to analyze the conserved domain structure. We obtained 20 motifs based on 17 sequences of VmGH18s, with sizes ranging from 13 to 50 amino acids (Figure 2b). In group A, motifs 5, 1, 9, 6, 2, and 3 constructed a conserved domain, and motif 13 was only distributed in this group. In group B, as shown in Figure 2b, motif 5, followed by motif 1 with functional domains, encoded glycosyl hydrolases of family 18, and motifs 16, 17, and 19 were only found in this group. In group C, motifs 5 and 1 followed by motifs 9 and 6 constructed conserved domains, and except VmGH18-8, the remaining class C proteins included motifs 12, 7, 5, 1, 9, and 6 and 2, 18, 3, 8, and 4, which constructed a conserved GH18 domain. Interestingly, motifs 5 and 1 were found in all the VmGH18 proteins and motifs 9 and 6 appeared in almost all the VmGH18 sequences except VmGH18-17. Some motifs such as motifs 16, 17, 19, and 20 were only found in genes clustered in the same group, indicating that these genes had similar functions. The specific construction pattern of the motif was the same as the phylogenetic relationship (Figure 2b).

### 3.2. Chromosomal Location of VmGH18

**Figure 3:**
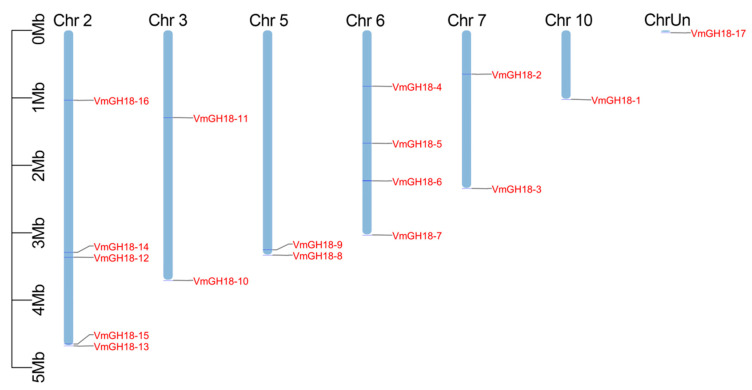
Chromosomal distribution of *VmGH18* genes. Tbtools was used to depict the chromosomal locations, and genes are marked with short lines.

The evolutionary relationships within a gene family are typically analyzed according to their chromosomal distributions. We determined the chromosomal location of the *VmGH18s* genes based on the genome database. The 17 *VmGH18* genes were unevenly distributed across seven cucumber chromosomes (Chr2, 3, 5, 6, 7, and 10 and ChrUn) of *V. mali*. Chr2 contained the highest number (5) of GH18 genes, while the lowest number (1) of *GH18* genes was found on Chr10 and ChrUn (Figure 3).

### 3.3. Gene Structure and Conserved Motif Analyses

The exon–intron structures were analyzed to explore the structural diversity of the *VmGH18* members. The numbers of exons and introns in the *VmGH18* gene family ranged from 2 to 16 and 1 to 15, respectively. Although some *VmGH18* genes with higher similarities were embedded within the same cluster, the numbers, distribution, and locations of the exons/introns were different. Notably, *VmGH18-2* and *VmGH18-10* were clustered in the same group and showed a high similarity, and *VmGH18-6* had the most complicated gene structure with the greatest number of exons/introns.

### 3.4. Conserved Domains and Active Site Analysis of VmGH18s

To locate the catalytic domain and active site in each VmGH18 protein, we generated a multiple sequence alignment and conducted a motif-based sequence analysis. After the multiple sequence alignment of VmGH18 was carried out using Clustal W 2.1, according to the sequences with different structures, the 17 VmGH18s were divided into three classes, and the members of a certain class exhibited higher identity. The Pfam analysis results showed that all the VmGH18 proteins contained a Glyco_18 and Glyco_hydro_18 domain, and this was a common phenomenon in chitinase family 18. It should be noted that there was a unique protein in class C (VmGH18-1) with the LysM domain. Only two members of class C (VmGH18-11 and VmGH18-15) contained chitin binding 1 domain 1 (ChtBD1).

**Figure 4:**
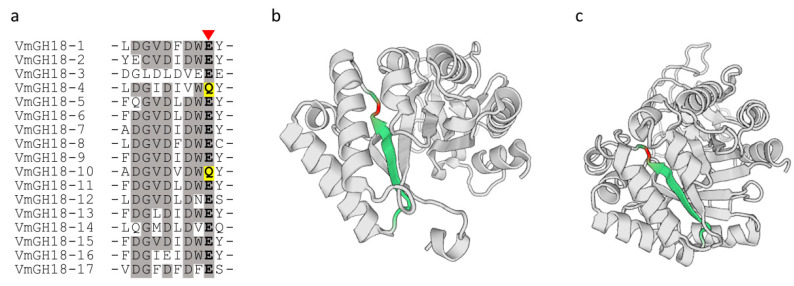
Multiple sequence alignment and structure analysis of VmGH18 proteins. (**a**) Multiple sequence alignment of the VmGH18s. Shading indicates amino acid sequence homology of 75–100%. Enzymatically critical amino acids are bolded and black, marked with red triangles. Mutation sites are highlighted with yellow background. (**b**,**c**) Structure analysis of VmGH18-4 and VmGH18-10. Green represents the critical residues from panel (**a**), and red represents the critical amino acids from panel (**a**).

Remarkably, the multiple sequence alignment of the VmGH18s showed that VmGH18-4 and VmGH18-10 were mutated at the catalytic motif of GH18 chitinases (DxxDxDxE) (Figure 4a). We identified 17 GH18-encoding genes in the *V. mali* genome, with VmGH18-4 and VmGH18-10 being the only two with substitutions in the catalytic glutamate (Figure 4b,c).

### 3.5. Gene Expression Profile of VmGH18

**Figure 5:**
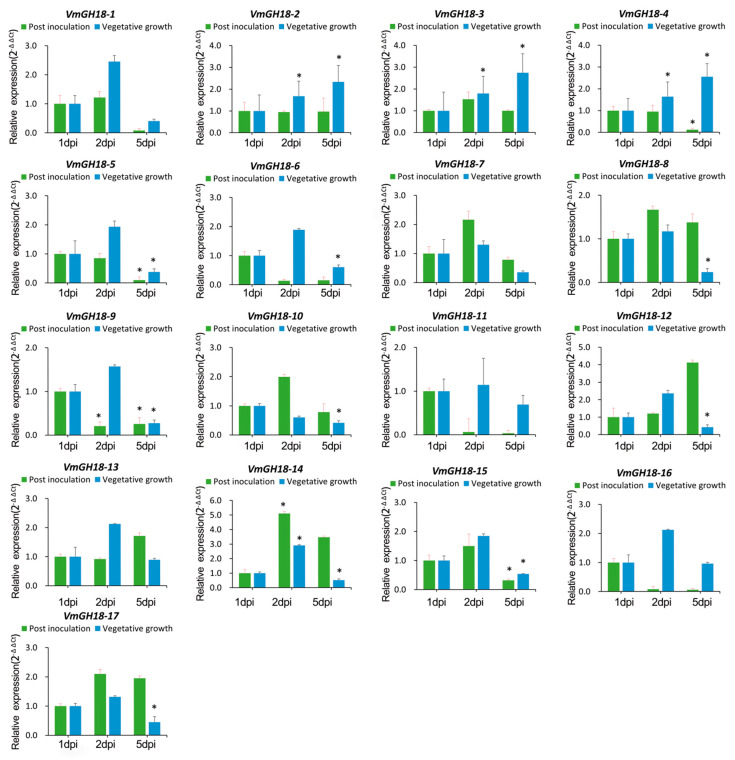
Expression patterns of chitinase genes in *V. mali* when infecting *M. sieversii* (green) and in normal growth on PDA plates (blue), as determined by qRT-PCR. G6PDH was used as an internal reference gene. The relative expression data were analyzed using one-way ANOVA to show significant differences, * indicates a significant difference at *p* < 0.05.

RT-qPCR was used to validate the transcriptional expression of 17 members within the VmGH18 gene family. RNA was extracted from samples taken at different infection times for *V. mali*-inoculated *M. sieversii*: 1 dpi, 2 dpi, and 5 dpi. Meanwhile, the vegetative growth fungal samples (1 dpi, 2 dpi, and 5 dpi) were also used for RT-qPCR, as a control for the results of the infected samples. All the VmGH18 transcripts of *V. mali* in infection could be detected and produced amplicons detectable by RT-qPCR (Figure 5).

After *V. mali* was inoculated on the *M. sieversii* leaves, *VmGH18-9* was downregulated on the second day, while three genes (*VmGH18-5*, *VmGH18-9,* and *VmGH18-15*) of *V. mali* were downregulated on the fifth day. Only *VmGH18-14* was upregulated on the second day. There were no significant changes for the other genes. Compared with the vegetative growth stage, three genes (*VmGH18-5*, *VmGH18-9,* and *VmGH18-15*) showed the same trend of downregulated expression on the fifth day post-inoculation. *VmGH18-14* was upregulated on the second day of the infection stage, which was the same as for the vegetative growth control, but this gene was downregulated on the fifth day for the vegetative growth stage. Three genes (*VmGH18-2*, *VmGH18-3,* and *VmGH18-4*) showed a sustainably upregulated trend for the stage of vegetative growth, and only the *VmGH18-4* gene was downregulated on the fifth day of the post-inoculation stage. Five genes (*VmGH18-6*, *VmGH18-8*, *VmGH18-10*, *VmGH18-12,* and *VmGH18-17*) were downregulated on the fifth day of the vegetative growth stage but showed no significant change for the post-inoculation stage.

### 3.6. VmGH18-4 but Not VmGH18-10 Is an Elicitor of Plant Cell Death

**Figure 6:**
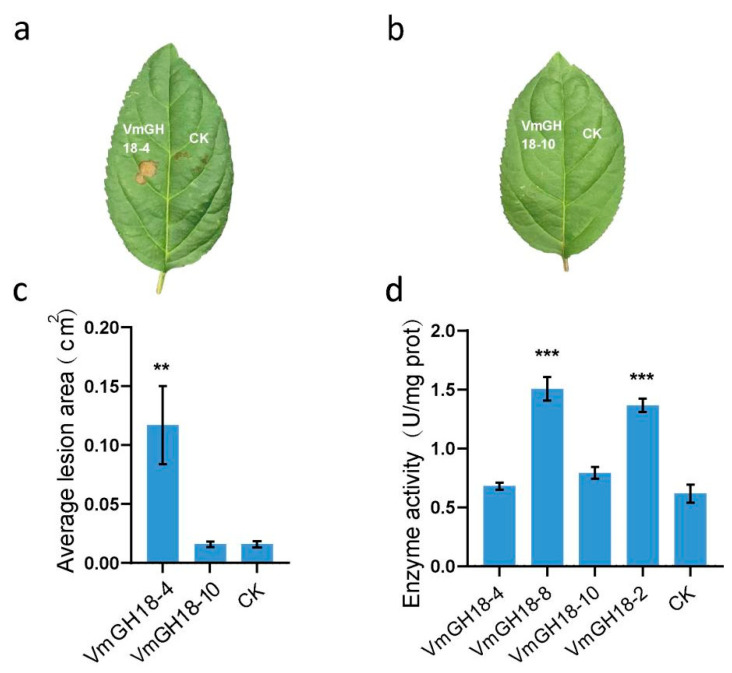
Functional characterization of two natural mutant chitinases. Cell death triggered by VmGH18-4 and VmGH18-10 (**a**,**b**). Cell death response in apple triggered by 20 µM purified VmGH18-4 and VmGH18-10 recombinant protein or buffer control. VmGH18-4 and VmGH18-10 proteins were dropped onto needle-pricked areas on apple leaves. Photographs were taken 2 days post treatment (dpt) for apples. (**c**) Average lesion area caused by purified VmGH18-4 and VmGH18-10 recombinant proteins. The lesion area was measured using ImageJ 1.8.0. (**d**) Enzymatic activity of VmGH18-4, VmGH18-8, VmGH18-10, and VmGH18-2. The relative expression data were analyzed using one-way ANOVA. ** indicates a significant difference at *p* < 0.01 and *** a significant difference at *p* < 0.001 according to one-way ANOVA test.

To examine the specificity of the plant response to the expression of VmGH18-4 and VmGH18-10, the recombinant protein was produced in *E. coli*. The purified VmGH18-4 (Figure 6b) recombinant protein retained cell-death-inducing activity, but VmGH18-10 could not induce cell death on apple leaves (Figure 6a).

To further validate whether the mutation sites in VmGH18-4 and VmGH18-10 affected their enzymatic activity, we utilized the chitinase assay kit from Solarbio (product number 9177) to measure their chitinase activity (Figure 6d). This kit measures chitinase activity by monitoring the release of NAG from chitin. The results conclusively demonstrated that neither VmGH18-4 nor VmGH18-10 possess chitinase activity.

## 4. Discussion

Chitinases play a role in fungal development and are characterized by the presence of the glycoside hydrolase 18 family (GH18) [50,51]. *V. mali* is a pathogenic fungus and major cause of canker disease, which can cause serious economic losses in apple production [52]. The chitinase gene family has been extensively investigated and identified in various fungal species, such as *Mycogone perniciosa* [53], *Mycoparasitic Trichoderma* spp. [54], *Blumeria graminis* f. sp. [55], *Saccharomyces cerevisiae* [18], *Trichoderma atroviride* [56], and *Candida albicans* [57]. High-quality genomic data are available for *V. mali* [58]. However, genome-wide identification of the chitinase gene family and its expression patterns have not been reported previously for *V. mali*. In this study, 17 putative chitinase genes (*VmGH18*) were identified in the *V. mali* genomes (Table 1). Based on their phylogenetic relationships and functional domains, the chitinases were classified into three classes (A, B and C) (Figure 1). Furthermore, *VmGH18* genes in the same group had a similar conserved domain and motif distributions to closely related members in the phylogenetic tree, revealing the functional similarity among proteins of the same subgroup. Gene structure analysis showed that genes in class A had more introns than those in classes B and C. A lower number of introns in a gene often tended to reflect faster gene regulation during the stress response; at the same time, introns also represent origin sequence variation [59].

The characteristics of the GH18 gene and its coding sequence were analyzed in detail. Isoelectric point prediction analysis showed that most of the potential chitinases are acidic enzymes, except for two genes that encode alkaline chitinase (Table 1). Previous studies have shown that the activity of chitinase hydrolysis sites is often associated with acidic amino acids [48].

The secretory extracellular GH18 protein plays a key role in pathogen toxicity and fungal cell wall remodeling [49,50], and also plays an important role as a key virulence target in the control of harmful fungi [51]. In this study, 9 of 17 VmGH18 proteins were predicted to have signal peptides (Table 1), indicating that they can be secreted outside the cell and may play a certain role in the pathogenicity of *V. mali*. The absence of signal peptides in proteins indeed suggests that these proteins may belong to the category of non-traditional secretory proteins. Unconventional protein secretion (UPS) involves the secretion of proteins that do not rely on the classical secretory pathway of the endoplasmic reticulum–Golgi (ER–Golgi). These proteins typically lack the typical N-terminal signal peptide sequence and are secreted into the extracellular space through alternative mechanisms [60]. The secretion of non-traditional secretory proteins mainly occurs in two ways: direct translocation across the plasma membrane (Type I) and secretion mediated by vesicular transport (Type III). In Type III UPS, proteins need to enter a vesicular carrier and are then transported to the outside of the cell through the vesicular transport system. Because these proteins lack signal peptides, how they enter the vesicular carrier becomes a key question. Therefore, the absence of signal peptides in a protein may serve as an indicator that it is secreted through non-traditional secretory pathways. In conclusion, the absence of signal peptides in some VmGH18 candidates is consistent with the possibility of unconventional secretion, which would allow these chitinases to act extracellularly without the canonical secretion pathway. This could have implications for their roles in pathogenicity and adaptation to the host environment.

A recent study showed that *Moniliophthora perniciosa*, fungal pathogens of cacao, express inactive chitinases (MpChi) in which the glutamate (E) that comprises the catalytic motif of GH18 chitinases (DxxDxDxE) is replaced by a glutamine (Q). However, these chitinases retain substrate-binding specificity and prevent host immunity [28]. Interestingly, in VmGH18-4 and VmGH18-10, the glutamate (E) in the catalytic motif (DxxDxDxE) is also substituted by a glutamine (Q) (Figure 4a). VmGH18-4 shared 30% of sequence homology with MpChi (Figure S4). Based on these data, we speculated that VmGH18-4 may function similar to MpChi. Despite the same amino acid replacement having occurred in the catalytic site, VmGH18-4 could induce cell death in the host while VmGH18-10 could not (Figure 6). Previous research reported that a chitinase, MoChia1, from *Magnaporthe oryzae* activated the immune response in maize. The mutation of glutamate (E137), the conserved amino acid in the enzymatic active site of MoChia1, to glutamine (Q) still elicits an immune response. These results imply that enzyme activity is not necessary for activating the host’s immune response [61].

To investigate expression, we analyzed the transcript levels of *VmGH18* genes during the infection of *Malus sieversii* and vegetative growth. *VmGH18-9* was significantly repressed during infection (Figure 5). A previous study reported that *VmGH18-9* was significantly downregulated during *V. mali*’s response to exposure to the endophytic actinomycete *Saccharothrix yanglingensis* Hhs.015 (Sy Hhs.015) [62]. In addition, the *VmGH18-4* gene was upregulated in the late stage of vegetative growth but suppressed during the infection (Figure 5). Overall, most of the *VmGH18* genes were differentially expressed on day 2 during infection or vegetative growth. Chitinase appears as a virulence factor in a variety of pathogenic fungi. By studying the structure and function of chitinase, specific inhibitors or pesticides can be developed. These inhibitors or pesticides can interfere with the activity of chitinase, thereby reducing the virulence and transmission of pathogens [63]. However, in our study, the contribution of chitinase to fungal virulence was not analyzed. In future studies, chitinase could be knocked out in *V. mali* to determine whether it affects fungal virulence.

In terms of application, plant immune inducers can activate or guide plant immunity [64]. Chitinases can be used to induce plants’ resistance to *V. mali* or inhibit the growth of pathogens, with the function of inhibiting viruses and protecting plants. In the future, chitinases could be developed and applied as innovative plant immune inducers.

In summary, a total of 17 GH18 genes were identified in the *V. mali* genome with variations in protein structure and physicochemical properties. Although the amino acids in the catalytic site have mutated in the same way, VmGH18-4 is functionally different from its ortholog in *Moniliophthora perniciosa*. Our findings may provide insights for understanding the strategies employed by *V. mali* to infect *M. sieversi* and may serve as a basis for further studies of *V. mali* pathogenicity.

## Figures and Tables

**Table 1:** Protein and gene structure analysis of 17 GH18 genes of highly pathogenic strain *V. mali*.

Sequence	Protein Product	Protein Length	pI	MW (kDa)	Signal Peptide	Genomic Position	Gene Length
VmGH18-1	KUI73695.1	1362	3.88	146.38	/	Chr11:10206981025079 (−)	4089
VmGH18-2	KUI71748.1	456	4.23	48.58	SP (Sec/SPI)	Chr7:646626648056 (−)	1371
VmGH18-3	KUI71641.1	349	4.52	38.3	/	Chr7:23421462343974 (−)	1050
VmGH18-4	KUI70699.1	278	8	29.73	/	Chr6:827947828966 (−)	837
VmGH18-5	KUI70476.1	404	4.17	44.39	/	Chr6:16707121672374 (−)	1215
VmGH18-6	KUI70319.1	1569	3.95	170.67	/	Chr6:22273132233150 (−)	4710
VmGH18-7	KUI70276.1	409	4.56	45.39	SP (Sec/SPI)	Chr6:30330583034434(+)	1230
VmGH18-8	KUI69933.1	265	4.53	28.94	/	Chr5:33309903332333 (−)	798
VmGH18-9	KUI69708.1	418	4	45.06	SP (Sec/SPI)	Chr5:32510833252716 (−)	1257
VmGH18-10	KUI67677.1	382	8.39	42.63	/	Chr3:37058953707093 (−)	1149
VmGH18-11	KUI67400.1	1183	4.68	127.29	SP (Sec/SPI)	Chr3:12911601294848 (−)	3552
VmGH18-12	KUI66967.1	338	4.32	35.75	SP (Sec/SPI)	Chr2:33626333363702(+)	1017
VmGH18-13	KUI66706.1	447	7.47	50.47	/	Chr2:46765024678403 (−)	1344
VmGH18-14	KUI66287.1	373	3.85	40.99	SP (Sec/SPI)	Chr2:32909873292234 (−)	1122
VmGH18-15	KUI66075.1	1145	4.85	123.78	SP (Sec/SPI)	Chr2:46476994651222 (−)	3438
VmGH18-16	KUI65769.1	602	4.08	64.63	SP (Sec/SPI)	Chr2:10324881034351(+)	1809
VmGH18-17	KUI64200.1	491	3.63	51.53	SP (Sec/SPI)	ChrUn:3131033037 (−)	1476

## Data Availability

The original contributions presented in this study are included in the article; further inquiries can be directed to the corresponding authors.

## Supplementary Materials

The following supporting information can be downloaded at: https://www.mdpi.com/article/10.3390/jof11040290/s1, Figures S1–S3. V. mali shows a highly conserved family of 17 VmGH18s protein sequences. Figure S4. VmCH18-4, VmCH18-10, and Mpchi show a highly conserved family of chitinase protein sequences. Table S1. Primers used in this study. Table S2. Phylogenetic tree and alignment proteins.

## References

[1] Synowiecki J., Al-Khateeb N.A. (2003). Production, Properties, and Some New Applications of Chitin and Its Derivatives. Crit. Rev. Food Sci. Nutr..

[2] Berini F., Katz C., Gruzdev N., Casartelli M., Tettamanti G., Marinelli F. (2018). Microbial and viral chitinases: Attractive biopesticides for integrated pest management. Biotechnol. Adv..

[3] Merzendorfer H., Zimoch L. (2003). Chitin metabolism in insects: Structure, function and regulation of chitin synthases and chitinases. J. Exp. Biol..

[4] Sánchez-Vallet A., Mesters J.R., Thomma B.P.H.J. (2015). The battle for chitin recognition in plant-microbe interactions. FEMS Microbiol. Rev..

[5] Horsch M., Mayer C., Sennhauser U., Rast D.M. (1997). Beta-N-acetylhexosaminidase: A target for the design of antifungal agents. Pharmacol. Ther..

[6] Prakash N.A.U., Jayanthi M., Sabarinathan R., Kangueane P., Mathew L., Sekar K. (2010). Evolution, Homology Conservation, and Identification of Unique Sequence Signatures in GH19 Family Chitinases. J. Mol. Evol..

[7] Fülöp L., Ponyi T. (2015). Classification of glycosyl hydrolases based on structural homology. J. Univers. Sci. Online.

[8] Gaber Y., Mekasha S., Vaaje-Kolstad G., Eijsink V.G.H., Fraaije M.W. (2016). Characterization of a chitinase from the cellulolytic actinomycete Thermobifida fusca. Biochim. Biophys. Acta BBA Proteins Proteom..

[9] Tian L., Weixing Z., Jing W., Yong Z., Yanwei D., Mingbo Q., Qing Y. (2018). The deduced role of a chitinase containing two nonsynergistic catalytic domains. Acta Crystallogr. Sect. D Struct. Biol..

[10] Suzuki K., Taiyoji M., Sugawara N., Nikaidou N., Henrissat B., Watanabe T. (1999). The third chitinase gene (chiC) of Serratia marcescens 2170 and the relationship of its product to other bacterial chitinases. Biochem. J..

[11] Karlsson M., Stenlid J. (2008). Comparative Evolutionary Histories of the Fungal Chitinase Gene Family Reveal Non-Random Size Expansions and Contractions due to Adaptive Natural Selection. Evol. Bioinform. Online.

[12] Benítez T., Rincón A.M., Limón M.C., Codón A.C. (2004). Biocontrol mechanisms of Trichoderma strains. Int. Microbiol..

[13] Gruber S., Vaaje-Kolstad G., Matarese F., López-Mondéjar R., Kubicek C.P., Seidl-Seiboth V. (2010). Analysis of subgroup C of fungal chitinases containing chitin-binding and LysM modules in the mycoparasite Trichoderma atroviride. Glycobiology.

[14] Yoshihito K., Toki T., Tomoyuki N., Takayuki O., Tamo F. (2022). Structure, mechanism, and phylogeny of LysM-chitinase conjugates specifically found in fern plants. Plant Sci..

[15] Eijsink V., Vaaje-Kolstad G., V?Rum K.M., Horn S.J. (2008). Towards new enzymes for biofuels: Lessons from chitinase research. Trends Biotechnol..

[16] Yamazaki H., Yamazaki D., Takaya N., Takagi M., Ohta A., Horiuchi H. (2007). A chitinase gene, chiB, involved in the autolytic process of Aspergillus nidulans. Curr. Genet..

[17] Shin K.-S., Kwon N.-J., Kim Y.H., Park H.-S., Kwon G.-S., Yu J.-H. (2009). Differential Roles of the ChiB Chitinase in Autolysis and Cell Death of *Aspergillus nidulans*. Eukaryot. Cell.

[18] Dünkler A., Walther A., Specht C.A., Wendland J. (2005). Candida albicans CHT3 encodes the functional homolog of the Cts1 chitinase of Saccharomyces cerevisiae. Fungal Genet. Biol..

[19] Kuranda M.J., Robbins P.W. (1991). Chitinase is required for cell separation during growth of Saccharomyces cerevisiae. J. Biol. Chem..

[20] Adams D.J. (2004). Fungal cell wall chitinases and glucanases. Microbiology.

[21] Seidl V. (2008). Chitinases of filamentous fungi: A large group of diverse proteins with multiple physiological functions. Fungal Biol. Rev..

[22] Li D.C. (2006). Review of Fungal Chitinases. Mycopathologia.

[23] Gan Z., Yang J., Tao N., Liang L., Mi Q., Li J., Zhang K.Q. (2007). Cloning of the gene Lecanicillium psalliotae chitinase Lpchi1 and identification of its potential role in the biocontrol of root-knot nematode Meloidogyne incognita. Appl. Microbiol. Biotechnol..

[24] Alcazar-Fuoli L., Clavaud C., Lamarre C., Aimanianda V., Seidl-Seiboth V., Mellado E., Latgé J.P. (2011). Functional analysis of the fungal/plant class chitinase family in Aspergillus fumigatus. Fungal Genet. Biol..

[25] Emri T., Molnár Z., Szilágyi M., Pócsi I. (2008). Regulation of autolysis in aspergillus nidulans. Appl. Biochem. Biotechnol..

[26] Pcdcsi I., Leiter c., Kwon N.-J., Shin K.-S., Kwon G.-S., Pusztahelyi T., Emri T., Abuknesha R.A., Price R.G., Yu J.-H. (2009). Asexual sporulation signalling regulates autolysis of Aspergillus nidulans via modulating the chitinase ChiB production. J. Appl. Microbiol..

[27] Ihrmark K., Asmail N., Ubhayasekera W., Melin P., Stenlid J., Karlsson M. (2010). Comparative Molecular Evolution of Trichoderma Chitinases in Response to Mycoparasitic Interactions. Evol. Bioinform..

[28] Lorencini F.G., Andrea S.V., Toledo T., Vital D., Costa D., Oliveira F., Thomma B., Guimar?Es P., Lima T. (2018). Suppression of Plant Immunity by Fungal Chitinase-like Effectors. Curr. Biol..

[29] Alhoraibi H., Bigeard J., Rayapuram N., Colcombet J., Hirt H. (2019). Plant Immunity: The MTI-ETI Model and Beyond. Curr. Issues Mol. Biol..

[30] Jaswal R., Kiran K., Rajarammohan S., Dubey H., Singh P.K., Sharma Y., Deshmukh R., Sonah H., Gupta N., Sharma T.R. (2020). Effector Biology of Biotrophic Plant Fungal Pathogens: Current Advances and Future Prospects. Microbiol. Res..

[31] Bruno Pok Man N., Pingtao D., Jonathan D.G.J. (2021). Channeling plant immunity. Cell.

[32] Lo Presti L., Lanver D., Schweizer G., Tanaka S., Liang L., Tollot M., Zuccaro A., Reissmann S., Kahmann R. (2015). Fungal Effectors and Plant Susceptibility. Annu. Rev. Plant Biol..

[33] Pusztahelyi T. (2018). Chitin and chitin-related compounds in plant-fungal interactions. Mycology.

[34] Jesús M., Diego R., Jesús H., Michael T., Antonio D.V., Alejandro P.G. (2021). Effectors With Chitinase Activity (EWCAs), a family of conserved, secreted fungal chitinases that suppress chitin-triggered immunity. Plant Cell.

[35] Abe K., Kotoda N., Kato H., Soejima J.I. (2011). Genetic studies on resistance to Valsa canker in apple: Genetic variance and breeding values estimated from intra- and inter-specific hybrid progeny populations. Tree Genet. Genomes.

[36] Ke X., Yin Z., Song N., Dai Q., Voegele R.T., Liu Y., Wang H., Gao X., Kang Z., Huang L. (2014). Transcriptome profiling to identify genes involved in pathogenicity of Valsa mali on apple tree. Fungal Genet. Biol..

[37] Feng H., Wang C., He Y., Tang L., Han P., Liang J., Huang L. (2023). Apple Valsa canker: Insights into pathogenesis and disease control. Phytopathol. Res..

[38] Meng X.-l., Qi X.-h., Han Z.-y., Guo Y.-b., Wang Y.-n., Hu T.-l., Wang L.-m., Cao K.-q., Wang S.-t. (2019). Latent Infection of Valsa mali in the Seeds, Seedlings and Twigs of Crabapple and Apple Trees is a Potential Inoculum Source of Valsa Canker. Sci. Rep..

[39] Liu X., Li X., Bozorov T.A., Ma R., Ma J., Zhang Y., Yang H., Li L., Zhang D. (2020). Characterization and pathogenicity of six Cytospora strains causing stem canker of wild apple in the Tianshan Forest, China. For. Pathol..

[40] Liu B., Peng L.X. (2011). Studies on Tissue Culture System of Malus sieversii. J. Tianjin Agric. Univ..

[41] Jaina M., Sara C., Lowri W., Matloob Q., Gustavoa S., Sonnhammer E., Tosatto S., Lisanna P., Shriya R., Richardson L.J. (2020). Pfam: The protein families database in 2021. Nucleic Acids Res..

[42] Chen C., Chen H., Zhang Y., Thomas H.R., Xia R. (2020). TBtools: An Integrative Toolkit Developed for Interactive Analyses of Big Biological Data. Mol. Plant.

[43] Ivica L., Supriya K., Peer B. (2020). SMART: Recent updates, new developments and status in 2020. Nucleic Acids Res..

[44] Bailey T.L., Johnson J., Grant C.E., Noble W.S. (2015). The MEME Suite. Nucleic Acids Res..

[45] Bateman A., Martin M.-J., Orchard S., Magrane M., Agivetova R., Ahmad S., Alpi E., Bowler-Barnett E.H., Britto R., Bursteinas B. (2021). UniProt: The universal protein knowledgebase in 2021. Nucleic Acids Res..

[46] Almagro Armenteros J.J., Tsirigos K.D., Sønderby C., Petersen T.N., Winther O., Brunak S., Von Heijne G., Nielsen H. (2019). SignalP 5.0 improves signal peptide predictions using deep neural networks. Nat. Biotechnol..

[47] Yin Z., Ke X., Huang D., Gao X., Voegele R.T., Kang Z., Huang L. (2013). Validation of reference genes for gene expression analysis in Valsa mali var. mali using real-time quantitative PCR. World J. Microbiol. Biotechnol..

[48] Livak K.J., Schmittgen T.D. (2002). Analysis of Relative Gene Expression Data using Real-Time Quantitative PCR. Methods.

[49] Punta M., Coggill P.C., Eberhardt R.Y., Mistry J., Tate J., Boursnell C., Pang N., Forslund K., Ceric G., Clements J. (2012). The Pfam protein families database. Nucleic Acids Res..

[50] Langner T., Goehre V. (2016). Fungal chitinases: Function, regulation, and potential roles in plant/pathogen interactions. Curr. Genet..

[51] Cecilia Gortari M., Alberto Hours R. (2008). Fungal chitinases and their biological role in the antagonism onto nematode eggs. A review. Mycol. Prog..

[52] Yin Z.Y., Ke X.W., Kang Z.S., Huang L.L. (2016). Apple resistance responses against Valsa mali revealed by transcriptomics analyses. Physiol. Mol. Plant Pathol..

[53] Yang Y., Sossah F.L., Li Z., Hyde K.D., Li D., Xiao S., Fu Y., Yuan X., Li Y. (2021). Genome-Wide Identification and Analysis of Chitinase GH18 Gene Family in Mycogone perniciosa. Front. Microbiol..

[54] Gruber S., Kubicek C.P., Seidl-Seiboth V. (2011). Differential Regulation of Orthologous Chitinase Genes in Mycoparasitic Trichoderma Species. Appl. Environ. Microbiol..

[55] Pham T.A.T., Schwerdt J.G., Shirley N.J., Xing X., Hsieh Y.S.Y., Srivastava V., Bulone V., Little A. (2019). Analysis of cell wall synthesis and metabolism during early germination of Blumeria graminis f. sp. hordei conidial cells induced in vitro. Cell Surf..

[56] Brunner K., Peterbauer C.K., Mach R.L., Lorito M., Zeilinger S., Kubicek C.P. (2003). The Nag1 N-acetylglucosaminidase of Trichoderma atroviride is essential for chitinase induction by chitin and of major relevance to biocontrol. Curr. Genet..

[57] Duenkler A., Jorde S., Wendland J. (2008). An Ashbya gossypii cts2 mutant deficient in a sporulation-specific chitinase can be complemented by Candida albicans CHT4. Microbiol. Res..

[58] Yin Z., Liu H., Li Z., Ke X., Dou D. (2015). Genome sequence of Valsa canker pathogens uncovers a potential adaptation of colonization of woody bark. New Phytol..

[59] Jan D., Van de Peer Y., Vanessa V. (2022). Nearby transposable elements impact plant stress gene regulatory networks: A meta-analysis in A. thaliana and S. lycopersicum. BMC Genom..

[60] Min Z., Lei L., Xubo L., Yang W., Ying L., Qing G., Shulin L., Yuxin S., Xiaoma T., Di Z. (2020). A Translocation Pathway for Vesicle-Mediated Unconventional Protein Secretion. Cell.

[61] Yang C., Yu Y.Q., Huang J.K., Meng F.W., Pang J.H., Zhao Q.Q., Islam M.A., Xu N., Tian Y., Liu J. (2019). Binding of the Chitinase MoChia1 by a Rice Tetratricopeptide Repeat Protein Allows Free Chitin to Trigger Immune Responses. Plant Cell.

[62] Liu C., Fan D., Li Y., Chen Y., Huang L., Yan X. (2018). Transcriptome analysis of Valsa mali reveals its response mechanism to the biocontrol actinomycete Saccharothrix yanglingensis Hhs.015. BMC Microbiol..

[63] Geum-Jae J., Fazlurrahman K., Nazia T., Young-Mog K. (2023). Chitinases as key virulence factors in microbial pathogens: Understanding their role and potential as therapeutic targets. Int. J. Biol. Macromol..

[64] Zheng J., Xu Y. (2023). A Review: Development of Plant Protection Methods and Advances in Pesticide Application Technology in Agro-Forestry Production. Agriculture.

