# Supplementary material for: Genome-Wide Identification and Analysis of Chitinase GH18 Gene Family in Valsa mali
Source: J Fungi (Basel). 2025 Apr 7;11(4):290. doi: 10.3390/jof11040290 (PMC12028287; doi:10.3390/jof11040290)
Supplement: Supplementary file 1 [file jof-11-00290-s001.zip › jof-3520870-supplementary.pdf]

Supplemental Figure Legends

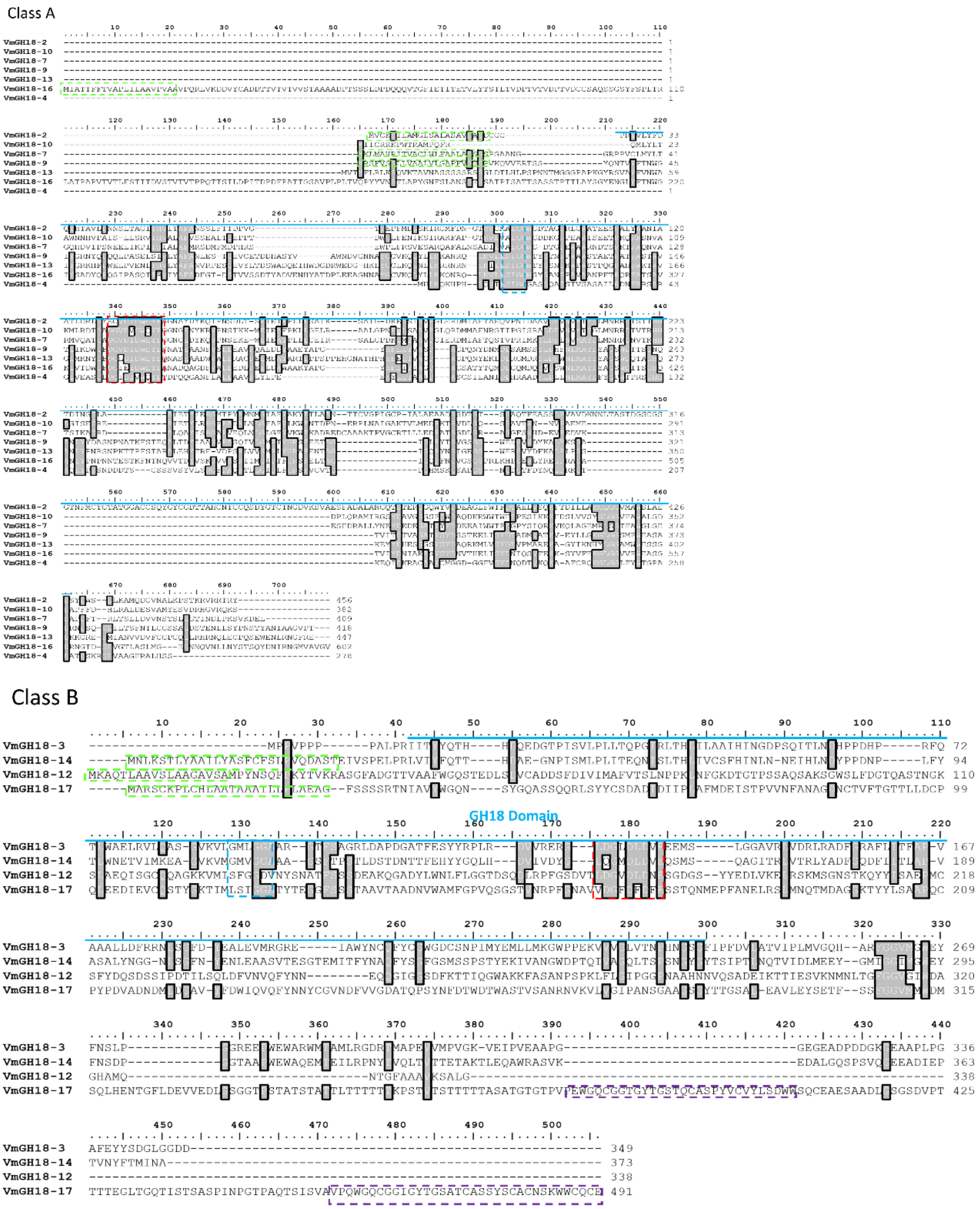

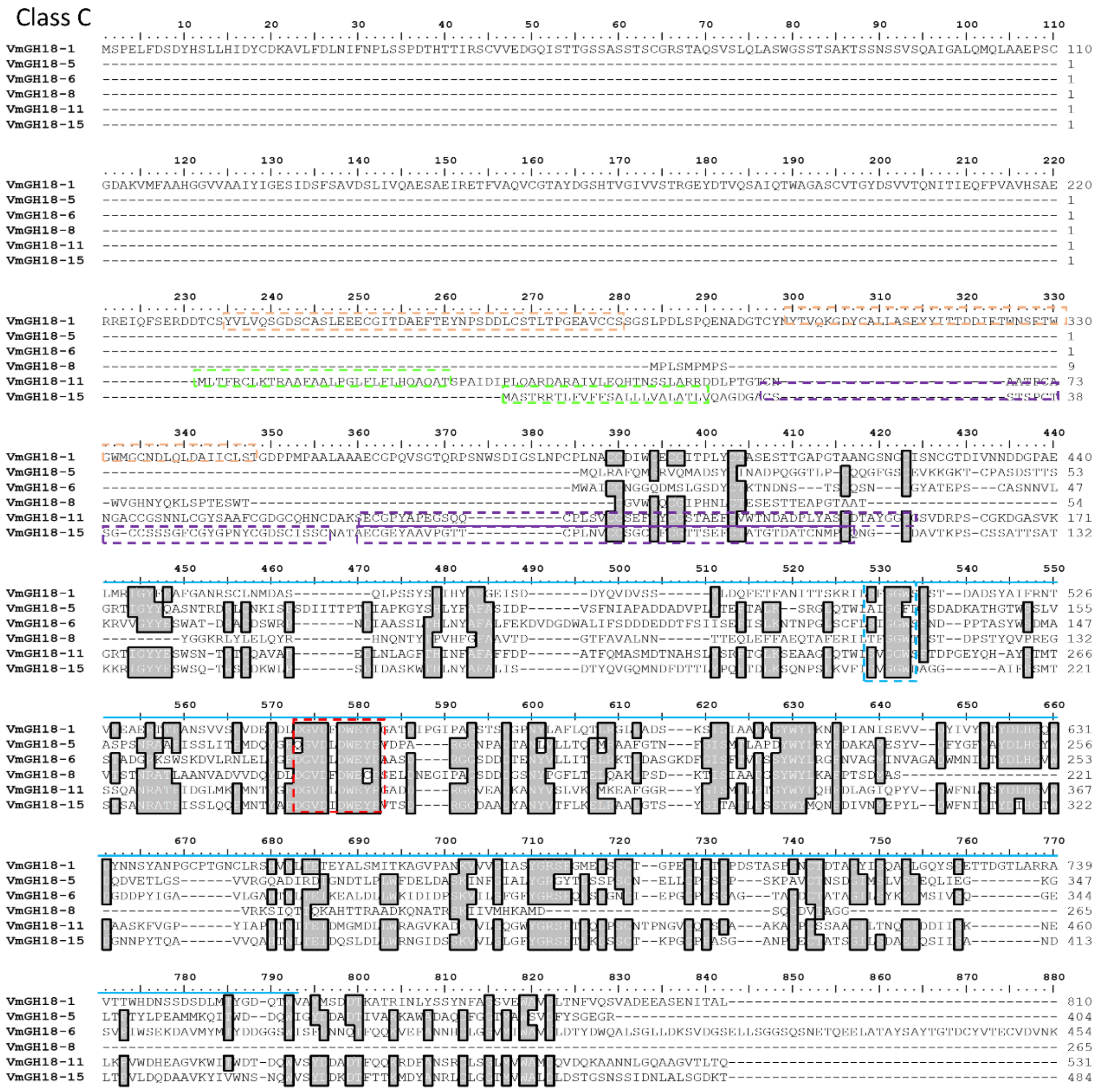

Figure S1-S3. *V.mali* shows a highly conserved family of 17 VmGH18s protein sequences. Three groups of *V. mali* GH18 proteins. A total of 17 genes encoding proteins belonging to the GH18 family were identified in the *V. mali* genome (03-8). Multiple alignments of GH18 chitinase subfamily Class A，Class B and Class C of *V.mali*. Amino acid sequences were aligned using ClustalW and visualized using BioEdit. Shaded amino acid sequences are 75–100% homologous. Blue blue line for GH18 catalytic domain. Green and purple dash line box represent the signal peptide domain and chitin binding domain, yellow dash line box represents the LysM domain. The amino acids in the blue and red dash line box indicate residues essential for Chitin binding site and catalytic activity site.

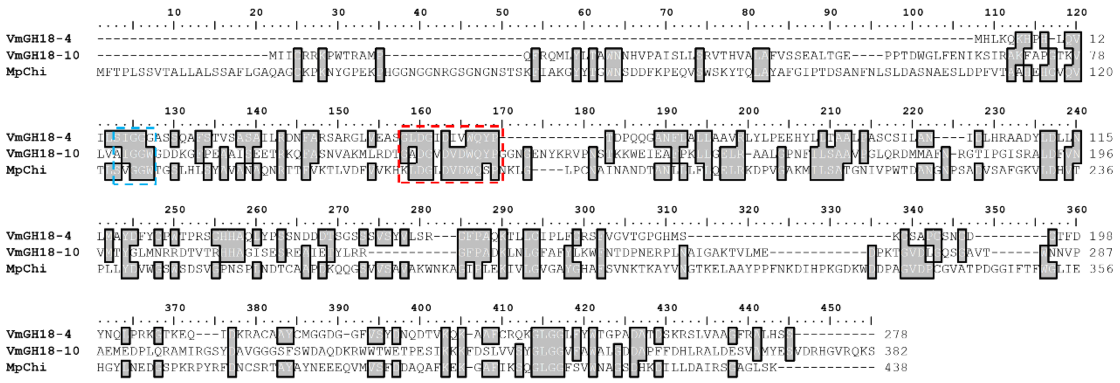

Figure S4. VmCH18-4, VmCH18-10 and Mpchi shows a highly conserved family of chitinase protein sequences. Proteins alignment of two species GH18 proteins. Amino acid sequences were aligned using ClustalW and visualized using BioEdit. Shaded amino acid sequences are 75–100% homologous. The amino acids in the blue and red dash line box indicate residues essential for Chitin binding site and catalytic activity site.

Table S1. Primers used in this study.

Gene ID	Protein ID	qPCR-Forward primer	qPCR- Reverse primer
VmGH18-1	KUI73695.1	GACTACTGCGACAAGGCTGT	GCGAGTTGAAGACTGACGGA
VmGH18-2	KUI71748.1	CCTGCTGGAAATGCCTACGA	ATGTTAGGGACTTGCTCGGC
VmGH18-3	KUI71641.1	CCTCCCACGCATCATAACCT	AGGGTGATTTGGCTTGGGTC
VmGH18-4	KUI70699.1	AACTTCCTCGCCTTACTCGC	GCCCGGTGGAGATCAATGTT
VmGH18-5	KUI70476.1	ACGAGCTTCTCTGTCCGTTC	CTCGGGCAGATACGTTGGAG
VmGH18-6	KUI70319.1	AGATGGGGTGCCGAGAAATG	ACATTCGTCGGCTCTTGGTT
VmGH18-7	KUI70276.1	CTCTGCTGACATCCTCGTCG	ACTTGCTTGTAGTCCTCGCC
VmGH18-8	KUI69933.1	GCCTTCCCAACTAGCGACAT	TAGACCTTGTCGCGTTCTGC
VmGH18-9	KUI69708.1	TAGCCTGGAATGATGTCGGC	TGTCCACCCACCGATACTGA
VmGH18-10	KUI67677.1	TTATCCTGTCTGCCGCTGTG	TCGCCTGTTCATCAATCCGT
VmGH18-11	KUI67400.1	CCGATAAACAGACCCGCCTT	TCCAGCATTTCTTGGCTCGT
VmGH18-12	KUI66967.1	TCGTCACAAGTCTCAACCCG	AGCCTGAGTGCCATCGAAAA
VmGH18-13	KUI66706.1	AAGAACTACGGCTTCGACGG	TAGGCGTCCATTTCTTCCCG
VmGH18-14	KUI66287.1	ACGGTTATGGCTACACCAGC	CGTCATCTCCTGTGCCCATT
VmGH18-15	KUI66075.1	CGCCACTCTGCGTCTATGAT	TGTCCCGTCACTATCCGAGT
VmGH18-16	KUI65769.1	CCTTGCCTCTTACCGTCCAG	CCAGAGTACGCAAGTGTGGT
VmGH18-17	KUI64200.1	CCCGTATGTCTGCGTCTACC	GCTGGAGTGCCTGGGTTTAT

Table S2. Phylogenetic tree and alignment proteins.

rotein ID	Species	Protein sequence
XP_386145.1	*F.graminearum*	MPPLPPSANPLPRLVTYYQTHHDSSGNLISTLPLITQPGIAITHVIVAAIHINEDPEKIALND<br>YHPSHPIFQTMWAELRILQASGVKVMGMLGGACKGSYARLDADENTFERFYCPVRDM<br>IRERALDGLDLDVEEVMSLGGIVRLIDRLRSDFGPTFIITLAPVAMSLIDFRRNLSGFDYEA<br>LEVMRGRDIAWYNTQFYCGWGDCSNPLMYDLMVQKGWPPEKIVIGLLTNPENGHGY<br>VPFNPLGMVLTTLRGRYGNFGGVMGWEYFNGLPGGKERPWEWAREMTELLRGHILS<br>EPAQVTQLTQQQQPVPVESAPKKNEEVDPDTPGGKDVALPKQFDYYTDGSDA
XP_388763.1	*F.graminearum*	MDRSPVPVTGAPVIQAFGGLNGYWGQLGSEPLKAYCDSHPEYITLSFVNQAPEQNPSN<br>LPGTNFAGHCAGGTYGNSNLLSECYTIQEGIPYCKDRGVKILLSIGGVYSDEGSNYKVTT<br>DDKGRDFADFLYKSFGPHNEHSNPIRPFDSIDNDGNTVHAAVDGFDFDIEHDLPNGPYI<br>AMINRLRELDEGLTITGAPQCPTSDEYFYMKDMIKSAKFDALFVQFYNNPWCDAVSQG<br>HEGESFNYDKWVEIIEESDCSKDAKLYVGLPASEEAAPGGGYLEPEDLKDLVCELTTKPHF<br>GGVSLWDLTRGAGNIIEGKSYNEHVMDALKYGCDLVSVPTTTTSAMTTTTEEASTSTNI<br>TTTEATTVHTTTTSDVSTTSGASSTEDATSTDITTASDVSSMEEATATSDVSASASTDATTS<br>MDATSTDTAASTDTAASTEAVASTYTTAATNSDASTSADVATSMDAATSTDATASADATA<br>STDVTTSSDASVSTDATASTETTATGANTSSVVTDPAASGVSATATESSADTATDSTTDSA<br>SKSDVLSATSDLTVTSDTSSVTGAASSSTIDAPVESDTATGTAATTNVSGATEPTTIATAYST<br>YRGWNTTSTGAAAYTQAYGNPTTLAHSGSLTTEGSPAYTNYPGNTGAVSMTTSTVCTT<br>RVHTVTQCPPEVVDCLFGSVTTETIPLYTTVCPVTDKAKHTGYATQYAPPPQYETRTVYT<br>TSVHAINKCAPGVVDCPYGSVTTETIPVYTTICPVTETYNQAPTDVPVNHEIKTLYTSQAH<br>TVAQCQPENPNCQVGAVTTEIASWTTAIVPAHETQLMKAYKPAAELPKPLTVESHLNGT<br>VYTSVVVPPATLKTATKPGVFEQARPTYKQTAQEQHSGAAAPTGGRNDKGPIETYAPAPI<br>TPATAGASSMVVGLTALAGIALLQVVALW
XP_389294.1	*F.graminearum*	MRLTWTTTLVALLLTATPFIVAEDIHCDKNKPCEVGCCGTNNVCGTGPDYCSKAKCINSC<br>TYKAECNPGKWDSQYFNAIKCPLNVCCSSYGFCGTTEEFCGKETVKRPSCSIGSQPVKR<br>VIGYYGSGGATHKCNPMIPDAFPQGIYTHIYFAFGSIDPKSFKVQPANAGDEQLYSQLSA<br>LKTRDSGQELWLSIGGWAFSDKGSPTATTFSDLVNADKIRQTYFFATLTLFMQTWGFTGI<br>DIDWEYPVDTDRNGRESDFKAYPRFLKRLKSALNNYKYGLSVTLTTSYWYLQHFDLDNIE<br>PSVDWFNVMSYDLHGAWDVGNKWTGAFVGAHTNLTEIKSSLDLLWRNKVSPSKVVL<br>GLAFYGRSVTLASSSCSEPGCPYLSAGDAGCSGEAGILFNSEISDLIREKKLRPKLYKDAAIK<br>TIQWNNDQWVSYDDRDTWKLKAKFLKSQCLSGVLVWAVDYDDDKHSYSNGLAAALG<br>IKVNVDSGSGLTIKLPDKTDEPKDFCYFTNCGQTCPSGYTEIVRGDKDSQIMMDGTECL<br>PGQDQVKTLCCPKSSKVPKCRWRGYQGNGHCPVGCNAGEIAVGTHHKGCEKSGWQT<br>ACCDATESTGLWSKCSWTDDCFDDNDSTCDKGFSTFVVGSRGGFGGQRTCKEGTKYN<br>YYYTETPKAFKDYEWVGHEVTFRNSQACSNAYPHRSTRIATQDISTTYSSSDNLAHTSNY<br>NYGFEAYCYAGDKESDKDERSVIEYQDRDAKEFDHALRKFLKDPVCPNEELQFGSDDGF<br>SGEPEFSPRSLVHGRATDNSADLEVLQHIMGVWFTSQNPRQDLTDIFNRDVEDAGYEG<br>QVANLTTLNRLLYQSDWNGQPRYSGRVVARMTLCNLAESREGLDNLATLPEALCKVPS<br>NTGSEAHRRDLQARTINVAVMANRGSGSTQPSAAYIFRAIELGHLSLHYTRWLPTAGQV<br>ILEVAYWIGRSPGYRDRTHVNAVDRWVMFHFHIPLNSNTFWTERNGVNDYYLGVSSF

XP_384928.1	*F.graminearum*	NMYHSQGTSQQLPYRNYRGQIDHRAEWRYSRSWNSGSFNTGRFTDYNRRREPFNCR<br>WPAPGTTTGPAFYLGRDYSNRLNDLRTAGRPTDLASLINEFGMRIHRAGIFRSSNVAM<br>MWPEAANGNLPHAGSSWGDEEYHPDLEAFHRNYDANGNRVNNVQNADP<br>MMLRLSSIAFMAFALCLLCLVDFSNGDATNHPHNSHVRMHRRSNVITKAPAWAKKITP<br>GAAHQNPAKAPPNPTEDLELSIDKSTNLDQYYENSKNASESSAGVTPNKSHHSRRSLEK<br>RGSGALYCKDGPCADDSCCGPEGICGYGPDFCGDGCTSNCDAVAMCGEYSEDGDMP<br>CGMNLCCSSSGWCGTTEVFCDNADPVHKTLPCQAGYGSCNIASSPSCAKGGGSTNGR<br>TIGYYQSWNVRTRKCDTKTPKQLDTTGFTHLFYSFAFIDPTSFKITPAHDDDVKQMREFT<br>DLSKGGKLQTWIAIGGFDMSDEDKPTHTTWSDMVSTKANRAAFIESVRDYMDEYGFQ<br>GVDLDWEYPGEPKRGGRKLADTRNFSMLLREMRAAYGDKYGISLTLAPDYWYLRWFD<br>AKAMEPYVDFFGFMAYDLHGFWDEDVRTLGKIIRGQADIREIGNNTIPLWFDGLDPKKI<br>NFGLAMYGRGYTVADKSCNGLGCSFVGPSEKGECTDSDGVMSLGEIKNLIKNKGVKSIY<br>LEEAMMKQISWDDQWIGYDDEETFAAKKAWADGYCFGGTMVWSIDFQASSSSSSGG<br>GLSGGTGNANTGNDGSDFPDIGGSHLTHKQVIDKLMDEKMPDCFSDKAYKDIKDKGKF<br>WYDSGAEQWTDKYISAQKDHSQWAQNLYRDIFKNDVTAADFACFQPGEECNFGKSC<br>ADFNKKGKGGLYYMFLSLQNFFNWYSQFIQEFDQNMSYSAGNVSVIRKGLSIGESNPS<br>ASIDILSILAAAFTIASGPAAPIAPLGGAFAGLGGLMSMYSATTGDDVTVGNDAIDAEGS<br>ASAAISNMRKAGIDSAKKLIAAVFGKQGHPQSDIPEAMLVGDSKYKNPVVRVFGWGG<br>WVRDSALGGLEELVTGMRSNMDKALLWQMARAWRGLYVVVRDDLPKPKCINPNNA<br>WDDEKGRCLDILAWYPKTDKKTFGGNKDIEFAWNSWGMDKLGTLRNAVACWENNG<br>GKIGSPKTSIGSLTSSSPYDSPCFFAMPVLKGNYSDISKGSLWLAGDFAGQEGQAGKLWP<br>KTRCEAYNRDMHYIKRYTKDCSDLDVEGDLA
XP_391293.1	*F.graminearum*	MSDIIQKTTELPIPLMLTPTGDVKIARIWAISYIGKSYLSPELLAPTADCRVPLQQLSHVGC<br>HLGLNRRGRLDTVAGLGRGATDDTDADVIQPKVCINGRIPLKEGIEVERAEIGDDLGICLT<br>LSGLVERDATVDETILRGTGFRSTCDFLTPWRVIPCNEKSLDPESWSISSPLQGLFCSSWP<br>NGGANHFEVRRFCSDATAADSTTCSATRRCAQGCCNKSGNCGFGPDYCGKSVCRSDC<br>DRLSECNPGFGSAWAQRDKCPLNVCCSKHGYCGTTKDFCGSKTVKCPNCRKVGNIPRV<br>VGYFEGWAKNRACEVFMPEQIPIGLYTHINFAFGTINPFTYVVEANDEEGKLMYERLIAL<br>KRRDRNLKIFLAIGGWTFNDPGRTHKVFSNLVNSEGNQQKFLVSLMSFMALHKFDGLD<br>LDWEYPVDKDRGGMESDYENFPKFMSNLKDLMEDGDRGLTITLPASYWYLQYFDIKKL<br>ERTVDFFNIMSYDLHGVWDQHANWTKPYLNAHTNLTEIDSALDLFWRNDIDPDKIVM<br>GLGFYGRAFMAKIASCVEPGCQFNGPANAGKCSAEKGILLNSEIEGVIKDWDLTPKLYKE<br>EAVKLITWGREWASYDDAETLKLKVNRAEERCLGGVMVWAITHDTRDAKYNLALADV<br>LGRNTTKGSLDQTEDAGKWVKTPYEQCRWANCREGCPKGWKTVPRSDSGARKGEQ<br>MFDETGCGGDGGHLFCCPADEDTPSMKVYGTCKWGEYPECDASPDCPYEDWSWPL<br>ASSSSGSGGGKCNDRKNELGTPIIGVQARNYCCKTDPDMRFIDCEVRRDIGPYPKDEPIY<br>GFCRSGCPSDRIFKRGSSSTDLVARDGKIQLVTSHDILTDLIALAGPAALLPKMRLEWNTA<br>MRAANYTYLQMTYISRYIRENWILEWEGPSDFAREILCQPAYWAKVIRAWALGDTDPST<br>GAMNCTYAICDVNGKCLEDDDEGEGPAGNERRHANLFGRHATHLLSHSHHHHRRHTL<br>QPRIVEPLEVTDPDDTTEKHEYAIEIPDNPTAEVIAQDEDNPLLKNVYQIWFPSMCWVP<br>KIRVKPFAERGARPVQVEHLVDKNILKKFFISSALGKLRSGTVSKYGPIPIAFWDRMEQID<br>LALEPGVPDLPGSEGGTYQRSWIMDRAFECLGSARNDQVFVLIEKIINDAKNKVFCEDR<br>TNGVSSIKEKLKWGPKTPDKVKQDKAMILVTRIRDGFAAFLYINSSRVQLRKIVRDIYLNF

		QVAENVYNKKYKNEADFKRVQLADYWVEWIVDYFDFISNKFKRDVRQQISEIRAILNGVNEPIVTEVLAHLASFADMMDKNPAQGRVNPKILDGIKDDDTEMGGT
XP_387668.1	*F.graminearum*	MHFLKMSFAWLLALLAMATAADSAEPFRCIMYLTGQHDVVPAKHQFKGVSHVVIAFMRSEFFNVDEQPDDYPMFTSVSDVRARVPQHTKVMVAIGGWGDTQGFEEAAKTYFSRKRWTRQVAAMVKATEADGIDVDWEYPGGNRDDYKEIPNSEREWEIEAFVSLLQELRAALGPEKILSAAVPGKEGDLMAFTTDTVPRIMKEVNFLNIMSYDLMNRRDNTTVHHSGVENSQEAVQRYIDRGASPSSVNLGLGYYVKWFMTEKCDPAKPVGCRTPILEDPETGADLGKTGGFSWHDEVPKDVAQSFSRARYDGKYDVDGSYYYWDEQELRWWSFDTTRSIQTKFERIVPQLKLGGVFAWGIGEDAPDFEHFLTTAEEVRKIREGDQVKDEL
XP_382530.1	*F.graminearum*	MHLSALLGLGAAAVTSASRNIIYYDQWHTKDLPSKDITSGVTHVMMSFANSSLFTTQPGGKYEPFQPLEKVRSLFDHDIKVCLAVGGWGDNAGFDEGVKTDRSRERFARNVASTLDRLGFDCVDIDWEYPGGNGQDYKQVPNSKKTYEIKAFPKLLKEIKKFIGEKELSIAVPGLERDMIAYLPTEAPLINKYVDFVNVMTYDLMNRRDHYTTHHVSIAGAARAIDKYISLGFPPSKLVLGIPFYAKYFMTKKGYTCTEPIGCPTELLENPEDGSDTGKSGSMTFEAANFAAAPTNLTTSTDATCGAGTFFKCPAGSCCAASGWCGSTPAHCGTGCQSAFGKCDGVDINNSFHKALKDGRTDTVNGGQWYWDSETRIFWTWDTAELIAQKIAFMAQTRGVKSVMAWALALDSNDWSHLKAMQQGFKDVNA
XP_384319.1	*F.graminearum*	MPTQYINAVYYPSWRCYKERPPSCLDISSITHIFYAFVGQVSSFKQHFQAIDDWADNEKMVDGEKGCLAAISKLKSQHPHIKTLVSIGGGSSSKEFPALAANKTARQTFARRISEFCVTHQFDGVDIDWEHPQTPEAGRNYVLFLQDIRNVMTHSQFLLTSALPTGEYCLKHINLPVVAHLLDFLNLMGYDFTGGWTDVCGHHAQLLPPSQNLNEVYPTLRKSCQRGVDFLIANGFPRHKIILGIPVYARYFGQARGPGHPFKGAGEIDYCDLPDEWVTNAEVDQSVAAASFVDNKSDKGFVSFDVPSTVSIKAGYAKALGLGGLFYWTGAGDRKGCESLVTAGWTALNSQ
XP_381289.1	*F.graminearum*	MSNTSTSLRTITLKDRPLLPKTIRVSDMLNQIFLIAPLLVQAALAVPSVEPRHQHLHGHMEHSHGTRTTMSTAVRRQVEQTIEAVPQFIPPKMPYALKPQTRVTKPTAAASNDDDDDNTVPGFIPPRMPFNSKAVQQKSTNEQASPQRLASVASVNNKKTTAKDTKDTKDKMEAKETKDKKDSKNTKSTKNTKNTNAQKLAQVQEDDDDKLPTFIPPRSPFRGLKKPQPTQAVPRDTVPEFIPPRNPWAQSKFNTRSTDSDDQDDPEETDAPDSDQRASRVDLLKRDGEDEDDEYFLEEGDDEFSPSDFPNLGEPEDDTVDASDQHSQDTPAANMSKQQGDPEEGINVQGEENTKNYFGGNFDDDDAENTPARLEARGVAKRNMLYFTNWGTYEGFNPENLPVKEITHVLYSFAKVNAKDGTVESSDPWADVQRTYPGDNGGGGNNAYGCVRQLYILKKQNRNLKVLISIGGFDGSPALASGVSTQNGRKRFISTAIKLITDWGFDGIDVDWEYPVNAQEARNYVLILNGLRKALDKYSQDYKLNYRFLLTVASPAGSSHYNTMDLKKMDPWVDAWHLMAYDYAGPWDSTTGHQANVFASRKSPLATKLSTDATLNDYIAAGVSPNKIHLGMPLYGRSFANTAGLGKPYDGVAGSPANLGVYLLKDLPRPGAVTTYNADLMASYTYDRKKRELVTMDDLKSAQAKAGYINERNLGGAFYWEARGDRSGSASVVAGVSRTLGTLERSNNLLKYPTSIYENIRNNRP
XP_391115.1	*F.graminearum*	MGGGPEGFRTVAYFVNWAIYARKHRPQDLPVENLTHILYSFANIRSDSGEVHLTDSWADTDIHWDGDSWNDVGTNLYGCMKQLNLLKRRNRNLKVLLSIGGWTFSSNFKGPASTPQGRATFAKSCVDLIKNLGFDGIDIDWEYPQNADEARNYVELLGAVRYEMDAYAQTLSRPYHFELTVACPAGATNFQKLDIRGMDRYLDFWNLMAYDYAGSWDQTAGHQANLYPSHDNPVSTPFSTSAAIDFYVRSGVSPSKIVLGMPLYGRAFQNTDGPGRPYQGVGEGTWEQGVYDYKALPLEGAQEYGDRGCCASYCYNPQTRTMVTYDTPRVAWDKAEYVRKWKLGGAMWWESSADKEGEKSLITTVVNGFGGQGALMRQDNCIEYPATKYDNLRDGFPNN

XP_390905.1	*F.graminearum*	MWLSVRRALAAVAISQATFCLALLPRLPPYTKEIAQQSKNPVNAVYFTNWGIYGRNFQPQDLPASEITQVLFAFLNVKPDGTVYTGDAYADLEKHYQGDRWDDQEENAYGCVKQLFLLKKAHRHLKVILSIGGWTWSTNFPSAAGTRENRIRFSKSAVTLMKDWGFDGIDVDWEYPNDENEATNFDLLLQAVRDELDSYASQNAPGHHFLLSIAAPAGPEKYKKLHLDKISNIVDQINIMAYDYSGSWDSASGHNANLFPYKASTNPYNSDKAINDYIDAGVPAEKIVLGMPIYGRSFEGNLGIGKSFSDVGQGSWERGVWDYKALPKPGAEIKYDEEAQAYYSYDSIMHELISYDTPEEVEKKVDYVLKHGLGGSMFWEASGDKKGNESLIGTSYNCLGTLDESENWLSFPDSRYANIALGMPGQWAQV
XP_383767.1	*F.graminearum*	MVAREDIPTNVPDLVVADLNGEDEVSEQATGSVNAVYFVNWGIYGRNYQPMNLPASQLTHVLYAFLNVRADGTVYTGDSYADLEKHYTGDSWEEPGTNAYGCVKQLFLLKKANRKLKVMLSIGGWTWSTNFPAAASTAATRATFAQSSVALMKDWGFDGIDVDWEYPANDTDANNMVLLLQAVRKELDTYSKQYASGYHFQLSIAAPAGPENYGKLKMKELGSVLDHINLMAYDYAGAWSAFSGHQANKYANAKIPNATPFNTDQAVSAYVGGGVPSGKMVLGMPIYGRAFQNTGGLGQGYSGVGSGSWENGIWDYKVLPKPGASLVYDRDAQASYSYDANTKELISFDTPGMVENKVLYVKNKSLGGSMFWEASADKTGADSLLGTSAKKLGSLDSTTNCLTYPNSRYANIAKGLN
EHK24036.1	*T.virens*	MFVRNAVAITGLLATLSNALPAKRALDAGNARLVIYWGAEDDSTTLSDVCSDDSYGIVNLAFLNRFFAAGGWPEISMSGLDNSSDAQQSAGATGLKDGSGLVDAIKQCQSAGKLVILSLGGADADVTLQSDSDGEKIADTLWNLFGGGTENAELRPFGDIKLDGFDLDNESGDSTGYLAMTQRFRSNFQSDTSKTYYLTAAPQCPFPDASEPLDVCKELDYVWVQFYNNGDCNIAQSDFKNSVQTWSSGIGNATLFIGALASGADGDQGYVDADTLVSSLQDVKNMNLPNYGGAMLWEAQLAVKNGNFQQKIAAGL
EHK21641.1	*T.virens*	MPWISLWTITWLLAWTITAVAASTPEHVSSDDYDATAFQSQFNRCPVPCLDESPEDWSVYSSFERLSQCDRPMLFDLAIHTPINDKKKSVLIRACTTSTSNKKSHHKSDAAKRELDACRDSFVQTKVQLHVSRDVDKPTTKPREGLIILFNKMKDYAQSIVATTGCSKESDVMLGYFDGSVVGIYAGRSLASATVPPMIDHLFDQYKYPGYATSSMLTQICGHESDMDHTAGVAVSLEADIDYVQSALLSWNQGECASKPNIADDGSTYRDLTIHQVPLPAALNSVAQGVENVNSGSKDLGRVTNMAEHLDAQVNGKYCTMHKIAVGDTCASIAKTCKISVANFLKYNDVKGDGNDFCRKLQAEKNICCSSGSSKPLPEDNGGCYTYTIKANDECSTIGAPWNLTPKDIEGFNNKITWGWRGCPNLTVGLKVCLSKGSPPMPAPISNAVCGPQKPGTVNPGQVKDASTLAKLNPCPLNSCCNIWGQCGIDSLFCTKADGPTGNPGTAPPRANGCISNCGTDIVNNDKPPSNGFKRVGYYEAFNWERSCSHMRSEMSNTQKYTHMHWAFGDVKSDMSVSINDTYGQWYGFMGLKDVKKIVSFGGWGISTGVESYEVLRKAMSPESRDRFISNVVSFAKEANVDGIDFDWEYPGAPDIPGIPKGLPSDGPNYLNTLRALRKALPARYSLSIAVPASYWYLRQWDVGNGWAMSGCPSGNCLRSHINQTEVVLALSMITKAGVNANKVFVGESSYGRSFRMSNAGCTGPDCTFTGANGQSNAAKGRCTNASGYLSNAEISEIISKSSKSKRTWFDKDTASDYLVYNDVEWVAYMSDKTKEIRRDKWKKLNFGGSVDWAVDLQEFNVADTVGPHGQYNNASCIQVFDNMIWDWLNPSIEAVAGCTNILQPSPLPTAVTLTAYTTITLQLGTSLSTTVVSASFSISEVSYQPFTFNESHISTFSSGQMLTYNPTPRITPNPVTIGIPTGWTVTGVGKATGGNALKEPSVSIIGLPQMTSTSSSDHGIGFLLPITWLPTVSYKIPSILTPKPPAPTELPDDDEHPIINAPTPPGVVDCKDNSCTKGQDCDNDKCLQGGDCYGESCVSAGKCKGKKCIRGGNCVGFHCQQGGICEGEACEKGGGCSKKGSGNCNSGECRGKGCFPRSKCSGAQCERVTIKPLPRPKGTPVRLPRPTCLFGCPKLPDCPFWDLSCNAPCGLIGCPPGRQPTAKACTTLQTGRDCTEFISSTQVQTKPTTSWSTTTRTRCENVVDCEVTDMTFTTTIKSSTEEPDPTYIAKIIEY

		EGMKLMKDKDLLASIGKDEDEFFSLLESPTTTTLEISTTSEITSTPSEEASSEPTQTPEVTC<br>GIALYPPLMWRLDIIDMTGSWVWDDEGKNLQSEIKGCGAMTGWEWFKRKDGTREC<br>RFNLPLIIKEGCVERAIASAGGPDIDCIFAFG
EHK21522.1	*T.virens*	MRTTTLAAAAIAVTGAMAKPRYLMYFDQWDTQNLPDRSVTAGVTHVTTAFAATTLFTS<br>GEQYEPFMPLDQIRALFDDGTKICMAIGGWGDTAGFSAGAQNKTTRRAYAKNVAATV<br>KRLGYDCVDIDWEFPGGNGQDYIQTPNSEKAWEIDAFPLFLQEIKSAIGGHIELSVAAPG<br>RVEDMIAYTPENVAKINHIADFVNVMTYDLMMRRMNTTTHHSGVANSLASVTTYIER<br>GLSPSKINLGFAFYAKYFTTAPGYNCTTPVGCPTAVLEDAQGNDTGLSGAITFEVSTYAGA<br>LEHAVANGIADEQLGGQWWWDSEKEIFWTWDTAEFAARKFKEIVVPKGLGGVFAWA<br>LAQDSYDWSRFKAMQAGVKAMQHKYIELGITI
EHK19372.1	*T.virens*	MVRSLAYVGALLAALPSARAGFNASSTQNIAVYWGQNSANQANSQQRLSTYCANAEI<br>DIIPIGFMNGISPVITNFANAGNNCTAFPDNANALDCPQIEEDIITCQKTYGKTILISLGGG<br>SYSQGGFSSASAATSAAQTVWNMFGPVNPNSTVDRPFGSAVVDGVDFDFESGVNNL<br>ATFATELRSLMDASASSANRKFYLSAAPQCVYPDYADNPALNGVVSFDFIMIQYYNNGC<br>GVSSYVPGATIQWNYNFDVWDNWAHTVSKNPNVKILLGIAANTGAASGYVSGTQLSA<br>VISFTKQYSSFAGIMMWDMSQLYENSGFLDQVVSDLAAPGSSPPATTSTGGSKPTSTSG<br>GSTGPTGGGGGTVPQWGQCGGEGYTGPTQCQSPYKCVVSSTWWASCQ
EHK18477.1	*T.virens*	MLATLSLASPVKNLRAEPTDLPRLIVYFQTTHDQNNNPISMLPLINEKGIALTHLIVCSFHI<br>NQGGVVHLNDFPPDDPHFYTLWNETITMKQAGVKVMGMVGGAAPGSFNSATLDST<br>NSTTFEHYYGQLRDTITNFQLEGMDLDVEQPMSQAGITRLVARLRADFGPDFLITLAPV<br>ASALENSSNLSGFNYNTLRTAQGSNIAWYNTQFYSGFGSMQSTSDYDRIVANGYTPDT<br>VVAGQLTTPEGAGWIPTSNLNTTIISLVNKYGQIGGVMGWEYFNSLPGGTSEPWEWA<br>QIVTEILRPNLVPDLRITQEDADRLQSAYAASVKATGKDKSFVKKPSVDYNAMVNA
EHK19035.1	*T.virens*	MAVSISRCLATLLLVAGTIAPAAASKPDDGSDTGLDAWRAANDRTTAPQACPSSCQESD<br>SDPSGSSSWFLFPDATSLITCNETMLLSFNIQETIVDGKQVPVTAIRGCKADYSFNSALSN<br>SPMVNDDVAAVCSTPNHNIVKTSVTIGMPASDTTGGNNVPLLDDLLSAGHQVQNYLS<br>TKAPSCTENVVTFGHSQSAVIGLYAGAEVYQHGVHADILSNFLQDLAEKQPFHGPQIVQ<br>LCPENGRGADYAVGIIAASINDLTLVQDTVRTWADGSCVSGNFENYMTVSLRVPEQIQG<br>SNNKTLSTSSLSEEAHSWKKSRLAVRANCKTTTVHPNDGCSAVAQRCGISQSDLQKFNP<br>AKNFCSTLANFCALKVGQPVCCTHGKLPDITPKPNKDGSCSVYTIKPDDGCTTIAASHGL<br>TEAKIEEYNKKTWGWNGCGVLFEKAQICLSTGTPPFPPSISNAVCGPQVPGTKQPPFGN<br>SDDWVKLNPCPLNVCCNIWGQCGTTDDFCVISNSTTGAPGTAAPKKNGCNLGISNCG<br>RDIIKGSPPAKTMRVAYYESWNSNRKCLHMDVDEIDTSKYTHIHFAFANVTSTFGIDISG<br>AQDQFNSFKAMSGDVKKIISFGGWDFSTKPGTFNILREATKAANRATFQSNVVAFIKQH<br>NLDGVDIDWEYPGAPDIPGIPAGDADAGKDYYETLSSLKTALGSSKSVSLAAPASYWYLK<br>AFPVKDLGAKIDYIVYMTYDLHGQWDYDNKWTSPGCPTGNCLRSHVNITETKDALSMI<br>TKAGVPSNKVVVGVSSYGRSFKMAQAGCTGPMCQFTGSPRVSNAAKGRCTDTSGYIS<br>DAEINDIIMFGNVNKQYTDAGSNILVYNDTEWVAYMDDDTKADREALYASYNFAGTSD<br>WAVDLQEYTDGTGYDEGYDPNYVAKINPNYYASCDSTYSSLDELKNNQTNIPPHCMDQ<br>YIVDVEIKILSDALDKYKDLVDHSYDDKFKIYEEYTAEQIPAQINSFMGNGHAGDFFSCAE<br>TGNRTCCSITTTCPTIYKDGPDGVDWLSTKVPNVTYTLTDSDGFYKAIDQSYGVEKDWIK<br>FGNTDVQLTNGCQFEADIRECQRKNDKWFWNYPQPADDIKVFNPKDVIGKSYDKTQD<br>LLERLRLLRAIGSLDAQLDMADVGDAAALPALTVAVAVDSMEKVVKAADKIKKEEREEM

		IANFIGGVLFFIPFVGEAVDASMVAIRSALEMIEVAGEAGLLAYSIVLDPDNAFMAVFSTL<br>AGAGLSRESWSKAANERRSMKDEDVAKLGSIKDDLDKINTVRGGMCKI
EHK21978.1	*T.virens*	MLFQTSILFLLSLLPSIVVVQAQSCSASNPCKTGCCSKFGFCGLGPDYCASKICVANCDRK<br>AECDPGGYGAAYVNHTTCPLDVCCSKWGFCVLTQEFCGDKKVTQPSCSTTFHKFERVV<br>GYYEGWAMQRECNVFNPEKIPLGVYTHLNYAFATIDPKTFEVLPATVEDQQDTYDRVT<br>WLKKRDPDLKVFIAIGGWTFNDEGQPTRNTFSNIANNPQNQKAFTKSLISFMSTYDFD<br>GVDLDWEYPEAPDRYLQHFEIKKMAKIVDFFNVMSYDLHGVWDMPNKWVGPYLNA<br>HTNLTEIKDALDLLWRNNIPYDKVNMGLAFYGRGFTAADPKCLTPGCRYASGSEPWMC<br>SHEVGIVFNSEISDIMQSQNATPVLYKDAAVKVLTYNTNQWVAFDDEETLGLKLNFAKS<br>KCLGGVMVWAVSHETENATYSTALGKLAQRSTTSLINIGIDNGVHQYERVQNHPQCK<br>WSNCGEVLAEFLLTSRPQGCPAGWSPIQRSDPNARNGEIMIDDQGCEGQGSHYLCGL<br>GFRAWNDEIAAASLGGGLGSVLCQTQVQSGYCEDLSDEPDNGFPEKRTETRDYPWTTS<br>VAPTAEPLFLNLFNEIYSQVFLFPGPGCTNFVLRVEDRWNQRRNGDLENVVTEHLVELQ<br>TVKMWYTAAIQGVLATELGVPALPNPAGPIDPSFFMVTLPRANGPFLFNPPPPPPTGGP<br>LLSTMEARLFNALGSVANWGSFLLADSELNCLKEKLWNDKTKHFIGETMMRDMARST<br>DSADANIALNSIRNLVVIVSFLNHPTVHNYMAAIVMDFRRELRIGERVHFGLTGTTVAAV<br>DSFTNYFGSLMTRIDTRVANWAARWLNVIDATWNGVNTPAGIQIRSASASLRTQAQN<br>AAVNTQGFFDPV
EHK21404.1	*T.virens*	MVPRSRGWFCPCSCISGSSDSDQDHHQHQHQPNQDNEYEIRPPNAGRSYMNGVYY<br>PNWLVYKGKTPATLDVDNITHVFYAFVGVHEDGSLRWFDEHADMVKEVDGEKGALAA<br>LAKLKRNSPRLKTIVSIGGGTGSKEFPALAASRDARETFARQARQFCDRHEIDGIDIDWE<br>HPKDAEQGRNYVKLLQECRNALPEEDYFLTTALPVGQYILKHIDLDAVSRLVNYINLMAY<br>DFTGSWTSVCGNHAQLHSPIGDLQYSHPELRICATDGIDYVLSRGFPSRKLALGIPAYARY<br>FPRAEGPGCSTESAGEMDYCEIPDQWVENAVVDEAAVAAWYVDSDRDKGYVTFDVP<br>RTVYMKGRYAVHKGLGGLFYWTGTGDKGDGLSLVAAGRRGLDSQ
VmGH18-1	*V.mali*	MSPELFDSDYHSLLHIDYCDKAVLFDLNIFNPLSSPDTHTTIRSCVVEDGQISTTGSSASST<br>SCGRSTAQSVSLQLASWGSSTSAKTSSNSSVSQAIGALQMQLAAEPSCGDAKVMFAAH<br>GGVVAAIYIGESIDSFSAVDSLIVQAESAEIRETFVAQVCGTAYDGSHTVGIVVSTRGEYDT<br>VQSAIQTWAGASCVTGYDSVVTQNITIEQFPVAVHSAERREIQFSERDDTCSYVLVQSG<br>DSCASLEEECGITDAEFTEYNPSDDLCSTLTPGEAVCCSSGSLPDLSPQENADGTCYNYT<br>VQKGDYCALLASEYYITTDDIETWNSETWGWMGCNDLQLDAIICLSTGDPPMPAALAA<br>AECGPQVSGTQRPSNWSDIGSLNPCPLNACCDIWGECGITPLYCTASESTTGAPGTAAN<br>GSNGCISNCGTDIVNNDDGPAELMRIGYFEAFGANRSCLNMDASQLPSSYSHIHYAFGE<br>ISDDYQVDVSSYLDQFETFANITTSKRILSFGGWSFSTDADSYAIFRNTVTEAERTTFANS<br>VVSFVDEYDLDGVDFDWEYPGATDIPGIPAGSTSDGPNYLAFLQTLRGLLADSKSISIAAP<br>ASYWYLKNFPIANISEVVDYIVYMTYDLHGQWDYNNSYANPGCPTGNCLRSHVNLTET<br>EYALSMITKAGVPANKVVVGIASYGRSFGMEDSSCTGPECLFTGPDSTASPGNCTDTAG<br>YISQAELGQYSSETTDGTLARRAVTTWHDNSSDSDLMTYGDQTWVAYMSDDTKATRI<br>NLYSSYNFAGSVEWAVDLTNFVQSVADEEASENITALEDDFTAALSLSDYDISNFTTYNLT<br>DLATRLVGWEGCEYNEDTFPILHSQRIIYSGWQQSWKIMNYMYNVAKSGINFNEAAAV<br>EYLGPPALVSSTQQTSFNNIFKNLATIQPGWGGWFAWKLHVRCDDPENLCPCGVGPIA<br>YTTNKDADSGLARINFCQQYFYLQTLDDKMVFATTSFPVETYANVANYLQNQATVWIH<br>ELLHIDWVSTASTDANIAHVTDVKIQARFKSGKEWVKVYGAAMAKILGRLGHNTGGW<br>TMKNADSITLYGFAKYIQNALGNIYPHLPLASQSPTGISTGNGDVVFSAEDLFTVYDNGTI

		IESANTTLLDEYSWSTAQGVCAKADDEDGDLDSSDILTIGTDFAVQSDFPSAYLSSWSSDTDCSGDYYSLEGHNVDSTTDSCLVLRGGGLGTDVTGADVSCRWFTDGGETWADCGTSTLTQPKSWRVKGGTCTAYDTDDCSSDGYEDAYTSAQGCHNYDSSLDDTETWVALQCGADVDLDSLLQPIGNGTAVTATRGATAAASASMSVSSPRVTTPTVSSASQQPSRVFAVVSSSL
VmGH18-2	*V.mali*	MVCKSILAMGLSALASAVSASDCGGPRYVLYFDQYHTAVLPNNSLTAGITHVITAFANSSLFTTDPVGTYEPFMDLSKIRGMFDNGTKVCMAIGGWGDTAGFRLGAATEESRALYAANIAATLDRLGYECVDIDWEYPAGNAYDYKQIPNSDLTSEIETYPLLLSAIKSAIGDKELSIAVPGLERDMIAFTAEQVPNIDAAVDFVNVMTYDLMNRRDTSTTHHTDINGSLAAIETYIERGMTPYKMNMGIAFYAKYFTLAANTTCVGPIGCPIALAEAADGSDTGTSGAQTFEASSLPVAVDKNNLTASTDGSCGSGTNFMCTGTATGGACCSQYGYCGDTTAHCNTGCQSDYGTCTNGDVKSVAESFADALANGQTDTERGGQWYVDDEAGLFWTFDTAELVSQKFTDILLAKGLGGVMAWSLAEDSYDWSLLKAMQDGVNALKPSTKRVRRTRY
VmGH18-3	*V.mali*	MPPVPPPPALPRIITYYQTHHDQEDGTPISVLPLLTQPGIRLTHLILAAIHINGDPSQITLNDHPPDHPRFQTLWAELRVLQASGVKVLGMLGGAARGTFSAGRLDAPDGATFESYYRPLRDVVRERGLDGLDLDVEEEMSLGGAVRLVDRLRADFGRAFLITFAPVAAALLDFRRNLSGFDYEALEVMRGREIAWYNCQFYCGWGDCSNPIMYEMLLMKGWPPEKVVVGLVTNPHNGSGFIPFDVLATVIPLMVGQHARFGGVMGWEYFNSLPGGREEPWEWARWMTAMLRGDRTMAPEVVMPVGKVEIPVEAAPGGEGEADPDDGKGEAAPLPGAFEYYSDGLGGDD
VmGH18-4	*V.mali*	MHLKQKHPHLQVILSIGGGASSQAFSTVSASAILRDNFARSARGLVEASGLDGIDIVWQYPTDPQQGANFLALLAAVRLYLPEEHYLLTAALPASCSILANIDLHRAADYLDLLNLMAYDFYGPWTPRSGHHAQLYPSSNDDDTSGSSSVSYVLSRGFPAQKTLLGIPLFGRSFVGVTGPGHMSKGSAPGSNGDGTFDYNQLPRKGTKEQIDKRACAAYCMGGDGGFVSYDNQDTVKQKAAFCRQKGLGGLFYWTGPADATDSKRSLVAAGFRALHSS
VmGH18-5	*V.mali*	MQLRAFQMGRVQMADSYCINADPQGGTLPCQQGFGSCEVKKGKTCPASDSTTSGRTIGYYQASNTRDRLCNKISPSDIITTPTDIAPKGYSHLYFAFASIDPVSFNIAPADDADVPLYTEFTALKSRGLQTWIAIGGFDFSDADKATHGTWSSLVASPSNRAAFISSLITFMDQYGFQGVDLDWEYPVDPARGGNPADTANLVLLTQEMRAAFGTNFGISMTLAPDYWYLRYFDAKAMESYVDFYGFMAYDLHGYWDQDVETLGSVVRGQADIRDIGNDTLPLWFDELDASKINFGIALYGRGYTLSSPSCNELLCPFSGPSKPAVCTNSDGIMSLVEIEQLIEGKGLTPTYLPEAMMKQITWDDQWIGYDDADTIVAKKAWADAQCFGGTMAWSVDFYSGEGR
VmGH18-6	*V.mali*	MWAICCNGGQDMSLGSDYCTKTNDNSTSCQSNCGYATEPSCASNNVLKRVVGYYESWATDRACDSWRPNDIAASSLTHLNYAFALFEKDVDGDWALIFSDDDEDDTFSIISEFISLKNTNPGLSCFLSIGGWSFNDPPTASYWSDMASTADGRKSWSKDVLRNLELYGFDGVDLDWEYPAASDRGGSDDDTENYVLLITELRKTLDASGKDFGISFTVPSSYWYLRGFNVAGMINVAGADWMNIMTYDLHGVWDGDDPYIGAVLGAHTNLTEIKEALDLLWKIDIDPSKVILGFGFYGRSFQLSDGNCIEPGCPFSGAGTAGDCTATAGILSYKEIMSIVSQGESVPIWSEKDAVMYMTYDDGGSWISFDNNQTFQQKVEFANNHCLGGVMIWAVDLDTYDWQALSGLLDKSVDGSELLSGGSQSNETQEELATAYSAYTGTDCYVTECVDVNKGQCKAGYSVLEYVHAGTYGTIKAPDTDTCKTGTNLESENSDAQYRLICCPTQSMPESCIWGGGNDNGLCTGGGSSFCGEGKYELIQDSWTERWGAEKCVSGARSLCCNSNTELELCSWTSCGGSCPSDKSYANKYETLWPGSNLKPRADECLQGDEDDQGELALFCCPAEDTYKNCEWLADDYCTDSCPSDKVLITQRQEVVNIAKEDDYYDCLKGYVKLCCDPPDATTDWPVDPAYLFEDAD

		EDDVSWYYNVEEDSNELATGDASEDPFAIVMIDGDESAYDESLVDQWSFLDSNDNELKRRGARLSSRDIFAFRNDTFENVVENYRIRCGSSSLYVNGTGCQSIFIGGARNTIVKMPTHIGAGPYARVISLEPINSSVNTSVKARADEQVYELTVDYHLAAAAEDNKGDVNFRVDYTNLLEYWSDVTDTPAKHRKRWFGSFSSWLKKVTTIVKDEKGALPLEYDETIKLFHFYKYCPATKIEMTLDLDSNIHVGLYSQYAYYFEGSILPTPSIINTYGYFSVQPAAAILMTLRGEATVQTNSGDVDIVSGLTLPGLSIKGLISIGPTFALTGSMDTSLSVSGEINAGISVAWDRTEIYFPQDADGEAATVDPTDLDGDGYPQTYEVSPTLDATISAQGNMALTLTPQVKFGISVLSGSLMEGYVTAGVTNTVSLGVNATASANIDGELQAGYCYWADYVYSVFLSADVSFAGGVLDWGDSVELLSPDAPVTLVEETCQTWSNDNSDIRRRSSSLVQNTTGSPCFGGLIECTTEEATSCAADSGTTSETRKRATYSNPPGLFYNCDWFPSVTLTNLNENTMTGNPYYTFIAGGYDPTTGTTNRDEACGGSNGVAAQCAQSKTLMWPTAVQSAASSGTADPALMNGYQDTISCDEFPFNASEEGGDGAEAACVYNAQQGYQSTINGLLLQIKDVNAGLFWKTKNWPSAAAKRIYTMSLVYSSTTGNNLGKYAGQYSSNNVVTITNVLGGLNLFGNSAYTVNKNAVCLADASGLRTDPVTRVQSYVVTTCIVEFDTTTTNKRNVDFDPGDPGNWQIKSARLSDDWKEDAVWFEDGTPLFDDQGQIQDIRLSQSAPVPGATTAKPTTSPRETPGVVE
VmGH18-7	*V.mali*	MKLMAHRITVACLWLFAALASSSSPGAANGGRPPVCLMYLTGQHDVIPSNEELIKPITHIALAFMRSDMFMDPHRSEWPLFRSVESARQAFALNSADILVAIGGWGDTGFEAAARNDTSRKTFAQNVARMVQATGADGVDIDWEYPGGNGEDYKQVPNSEKEWEIEAYPLLLGEIRSALGPDKLMTAAVPGIERDMIAFTQSTVPRIMKYLDFLNVMAYDLMNRRDNVTKHHTSIKASRDALQAYISRGAPVEQLNLGLGFYVKWFKADREDCAAAKTPVGCRTLLLEDPATGGDLGRAGGFSWHDEVPEDVKESFDRALLYNEYDEDEGATYYYDDEEAIWWTFDGPYSIQRKVKQLAGEMRLSGTFAWGLGEDAPDFTRLTSLLDVVNSTSLLGTINDLPKSVKDEL
VmGH18-8	*V.mali*	MPLSMPMPSWVGHNYQKLSPTESWTCGVWGQCGIPHNLCTESESTTEAPGTAATYGGKRLYLELQYRHNQNTYTPVHFGFAAVTDGTFAVALNNTTEQLEFFAEQTAFERILTFGGWTFSTDPSTYQVPREGVTSTNRATLAANVADVVDQYDLDGVDFDWECPSELDNEGIPAGSDDDGSNYPGFLTELQAKLPSDKTISIAAPGSYWYLKAFPTSDMASVRKSIQTTQKAHTTRAADKQNATRSKIIVMHKAMDSQGDVTAGG
VmGH18-9	*V.mali*	MRSFVSSLLVAALVLGAPFVSTSPVKQVVERTSSYQNTVYFTNWGIYGRNYQPQQLPASELSIVLYAFANLESTGEVCETDDHASYVAWNDVGNNAYGCVKQLYLLKKANRQLKVLLSIGGWTYSTNFPAAASTAETRATFASTAVTLIKDWGFDGVDIDWEYPSNATEAANFVSLLEAVRQALDDYAAEYAPGYHFLLTVASPAGPQNYDNMDLSAMSSYLDHFNLMAYDYAGSWDTTSGNQANLYYDASNPNATKFSTEQALTDYIAAGVPASQIVLGMPIYGRSFEETAGIGLPYTGVGSGSWEDGVWDYKALPKSGATVITDTVAGATYSYDSSTEELISYDTADMVATKVEYLLGKGLGGSMFWEASADRNDSQSLILTSFNTLGGSSALDSTENLLSYPNSTYANIAAGVPT
VmGH18-10	*V.mali*	MIICRRKPWTRAMPQFRQMLYLTAWNNHVPAISLLSRVTHVALAFVSSEALTGEPPTDWGLFENIKSIRAKFAPGTKVLVAIGGWGDDKGFPEAAISEETRKQFASNVAKMLRDTGADGVDVDWQYPGGNGENYKRVPNSTKKWEIEAFPKLLGELRAALGPNFILSAAVPGLQRDMMAFNRGTIPGISRALDFVNVMTYGLMNRRDTVTRHHAGISESREAIETYLRRGFPADKLNLGFAFYLKWFNTDPNERPLNAIGAKTVLMEDPKTGVDLGQSGAVTWNNVPAEMEDPLQRAMIRGSYDAVGGGSFSWDAQDKRWWTWETPESIKKKFDSLVVSYGLGGVFAWALGDDAPFFDHLRALDESVAMYESVDRHGVRQKS
Vm	*V.mali*	MLTFRCLKTRAAFAALPGLFLFLHQAQATSPAIDIPLQARDARAIVLEQHTNSSLARRDDLPTGTCNAATPCANGACCGSNNLCGYSAAFCGDGCQHNCDAKSECGPYAPEGSQQCPL

GH18-11		SVCCSEFGYCGSTAEFCVWTNDADPLYASCDTAYGGCGSVDRPSCGKDGASVKGRTIGYYESWSNTRTCQAVAPEDLNLAGFTHINFAFAFFDPATFQMASMDTNAHSLYSRFTGLKSEAAGLQTWISVGGWSFTDPGEYQHAYSTMTSSQANRATFIDGLMKFMNTYGFDGVDLDWEYPGADDRGGVEADKANYVSLVKEMKEAFGGRYGISMTLPTSYWYLQHFDLAGIQPYVDWFNLMSYDLHGVWDAASKFVGPYIAPHTNITEIDMGMDLLWRAGVKADKVVLGQGWYGRSFTLQDPSCNTPNGVCQFSGAAKAGPCSSAAGILTNQEIDDIISKNELKPVWDHEAGVKWITWDTDQWVSYDDADTFQQKRDFANSRCLSGLMVWAMDQVDQKAANNLGQAAGVTLTQQQDAQQASADQQAKTTCRYGDCGASCPSGSTEVTESRGQPGQLSTTKDQCAAGSYRPLCCDGGTTMGTCVWRGWRGAGLSCMGGCDDGETEVTTNTNSHDKDTGHDQTCNGGLQTYCCKDFKPSTSKSSLLDDAEDAAKEAAEAAAEQAALDIAAKAFCRVAVPALLAPLEALEALIPIFGEIADIAEIAATPALIIACEKGIEKEGKAVFKVFGKEHSLSIDKPTAKPSTISERPPTTSHNPPKTSTKSDSCSRQARRDGELEARAACDKGTITRYTATDLHTPSVVKQCPVAASQACWHYYSAISRTPAWARQTCLETVVNRNFGGPVYATSKWSAEHLKQAEWVKWMARPRGGCERDEFPPAYFWEGAGAGQLIRYNPEAENNLGGKMWQKFCPETAAFSCRAGSEHDITPAGRGSITRECVKALTLSVLSIDFDDFLEDIPLAQEADKGLVANECYPRMPANPPANPEYFALLNADPTVNNLNAALWAGPVPASMRGGQNPPYRLRRRDGGGMHLDRDETGYDVDTALDLFVDAGNSSRPATPDERAAWEVDMSMAHQLLLDEEAARKGLEQVGYVNCLGDSCATQVTPINRPALATVEPAPTGPPVVEATTTTASAARASAAVVGTLGFNAAPSRPTTSQEMLELRS
VmGH18-12	*V.mali*	MKAQTLAAVSLAAGAVSAMPYNSQFPKYTVKRASGFADGTTVAAFWGQSTEDLSDVCADDSFDIVIMAFVTSLNPPKLNFGKDTGTPSSAQSAKSGWSLFDGTQASTNGKSLAEQISGCQQAGKKVMISFGGDVNYSNATFSSSDEAKQGADYLWNLFLGGTDSQDLRPFGSDVTLDGVDLDNESGDGSYYEDLVKELRSKMSGNSTKQYYISAEPMCSFYDQSDSSIPDTILSQLDFVNVQFYNNEQQGIGGSDFKTTIQGWAKKFASANPSPKLFLGIPGGPNAAHNNVQSADEIKTTIESVKNMNLTGFGGVGIWDAGHAMQNTGFAAAVKSALG
VmGH18-13	*V.mali*	MVTMFLRLKSQVKTAVNASSSSSRSSGLDTLHLPSPNNTMGGGPAPKGYRSVAYFVNWAIYGRKHFPWELPVENLTHVLYAFANVRPESGEVYLTDSWADQEIHWDGDRWEDGHKLYGCLKQLNLLKRRNRNLKILLSIGGWTYSSNFKAPASTPQGRAHFAKTAVGLMKNYGFDGLDIDWEYPQNASEAADWVALLRACREEMDAYARTLPPSPPEHGNATHPHHFELTVACPAGPQNYEKLDLRGMDRYLDFWNLMAYDYAGSWDQTAGHQANLYPNRSNPKTTPFSTARALEHYTRFVDPSKLVVGMPLYGRAFENTAGIGQPYNGIGEGSWERGVYDFKALPLPGSKEYYDHESGGSYSYDAQRKMLVSYDTVPMAREKAGYIKNHYLGGAMWWESSGDKKGRESMIANVVDVFGGPGQLLRHRNQLECPQSEWENLRNGFRE
VmGH18-14	*V.mali*	MNLKSTLYAAILYASFCFSLPVQDASTEIVSPELPRLVIYFQTTHDAEGNPISMLPLITEQNISLTHLIVCSFHINLNNEIHLNDYPPDNPLFYTLWNETVIMKEAGVKVMGMVGGAAAGSFTPSTLDSTDNTTFEHYYGQLHDVIVDYGLQGMDLDVEQSMSQAGITRLVTRLYADFGQDFIITLAPVASALYNGGNLSGFNYENLEAASVTESGTEMITFYNAQFYSGFGSMSSPSTYEKIVANGWDPTQIVAGQLTSPSNGYGYTSIPTLNQTVIDLMEEYGMIGGIMGWEYFNSDPGGTAAPWEWAQEMTEILRPNYTVQLTVTTETAKTLEQAWRASVKEDALGQSPSVQGEEADIEPTVNYFTMINA
VmGH18	*V.mali*	MASTRRTLFVFFSALLLVALATLVQAGDGACSSTSPCTSGCCSSSGFCGYGPNYCGDSCISSCNATAECGEYAAVPGTTCPLNVCCSGCGFCGTTSEFCTATGTDATCNMPCQNGCDAVTKPSCSSATTSATKKRIGYYESWSQTRSCDKWLPSDIDASKWTHLNYAFALISDTYQVGQMNDFDTTLYPQFTDLKSQNPSLKVFISVGGWDAGGAIFSSMTSTSANRATFISSLQQF

-15		MNTYAFDGVDIDWEYPVTSDRGGDAADYANYVTFLKELRAALGTSYGITATLPSSYWY<br>MQNFDIVNMEPYLDWFNIMTYDIHGTWDGNNPYTQAVVQAHTNLTEIDQSLDLLWR<br>NGIDSSKVVLGLGFYGRSFTLKDSSCTKPGCPFASGANPGECTATSGILSDAEIQSIISAND<br>LTPVLDQDAAVKYIVWNSNQWVSYDDKDTFTTKMDYANRLCLGGTMVWALDLDSTG<br>SNSSIDNLALSGDKTTGTSIERLTAVSRSNSMSLGLFWTVCLPKDTTSPCPQGFRAIAWG<br>HGKVFDADLQYNTGEGCHGGGVNGFQRALCAANNVLFDSIQWGPGSASKACNSKCP<br>NNWLTLTKNSHITGQKTGCKSGKYAPLCVYDMRALYTSNTCNTNAAGQLLSGGLSLRE<br>DDSGTADFDYDDSDSDGTALRVRQHREIQARKDERRQGQIQKRGFLSGGGCLGALPKG<br>DIALEVPATQLGVWDSGTTFYFFEPTSVKPSSSTKQSKTTSVEVVDTTTTTYPVVTRTCD<br>GDKYPQACYHYSSVAQMSTYSRATCSNLDNSNGLRPLTKSWNDGHKSYSSWNKYIAKS<br>YVNPNNKRKAARCQRDEWPPAHFQQGRADGWVRFLPGDQNGGIPNDKEGGWQGI<br>CRFPPKKEVVKQGGPITDMGNYYLMTTYTSTIITLNVMSYTWKNVNPPAGDPYGLTAN<br>VCRPSVLTTDVGYALQTDDPWYGGRRVSAYNKGPGSLTIGKSQPTYKRNKRGQEDGM<br>VVLDFNEDDEGDGLRVTADDGNSTRLATDEEIEELGYVRCNTPDCWEELEELRQMQAE<br>ALMATEAAGVIGSATDLVPASATETATVVGASTTGLEATRPTLVAYSGTQKTSTGSGPLP<br>METERGGGNGIGSRNHRRLHGQIRPHMVDLGTEDELAPV
VmGH18-16	*V.mali*	MIATTFFTVAPLILAAVPVAAVPQRLVKDDVYCADDTTVTVTVVSTAAAADPTSSSLDPD<br>QQQVTGFIETITETVLYTSTLTVDPTVTVDPTVDGGSAQSSGSYFSPLTRLATPAPVTVTLF<br>STITDVSTVTVTPPQTTSILDPITDPDFPATTGSAVPLPLTVQPYYVNSTLAPYGNFSLANS<br>STSSATPISATTSASSTPTTLAYSGYENGLYFTNWGIYSADYQPQSIPASQITRVIYAFADFG<br>TDGTVVSSDTYADVENHYATDPLEEAGNNAYGCVKQLYLLKKQNRQLKVLLSIGGWSYS<br>ANFPAVAANPFTRQRFATSAVKLVTDWGFDGIEIDWEYPANADQAGDFVALLEDLREEL<br>DAWAAKYAPGYHFLITIAGPAGSATYTQMDLGQMDQYVDSWNLMAYDYAGSWDITS<br>GHQANLYPNPNNTESTKFNTNQVVTDYVSKGVVASKIIMGIPTFGRSFELTAGIGQPFN<br>GVGSGSPRLKHPGEWLYRELPRAGATVIYDNIAKASYSYDNVTHELISYDNIQSVTEKKSY<br>VFEKGLGGVVFWEASGDRNGTDSLVGTLASLMGELNNQVNLLNYSTSQYDNIRNGM<br>VAVGV
VmGH18-17	*V.mali*	MARSCKPLCHLAATAAAILLPLAEAGFSSSSRTNIAVYWGQNSYGQASSQQRLSYYCSD<br>ADIDIIPLAFMDEISTPVVNFANAGDNCTVFTGTTLLDCPQLEEDIEVCQSTYGKTIMLSI<br>GGATYTEGGFSSSTAAVTAADNVWAMFGPVQSGSTVNRPFGNAVVDGFDFDFESSTQ<br>NMEPFANELRSLMNQTMDAGGKTYYLSAAPQCPYPDVADNDMLDGAVYFDWIQVQ<br>FYNNYCGVNDFVVGDATQPSYNFDTWDTWASTVSANRNVKVLVGIPANSGAAGSGYT<br>TGSALEAVLEYSETFSSFGGVMMWDMSQLHENTGFLDEVVEDLGSGGTPSTATSTATT<br>LTTTTTTKPSTVTSTTTTTASATGTGTPVPEWGQCGGTGYTGSTQCASPYVCVYLSDW<br>WSQCEAESAADLGSGSDVPTTTTEGLTGQTISTSASPINPGTPAQTSISVAVPQWGQCG<br>GIGYTGSATCASSYSCACNSKWWCQCE
MpChi	*Moniliophthora perniciosa*	MFTPLSSVTALLALSSAFLGAQAGCKPKNYGPEKPHGGNGGNRGSGNGNSTSKFIAKG<br>YYTGWNSDDFKPEQVSWSKYTQLAYAFGIPTDSANFNLSLDASNAESLDPFVTAAHEH<br>GVQVTLSVGGWTGSLHLSYAVANAQNRTTFVKTLVDFVVKHKLDGLDVDWQSPNKLG<br>LPCNAINANDTANLLLFLQELRKDPVGAKMILSATGNIVPWTDANGNPSADVSAFGKVL<br>DHVTPLLYDVWGSWSDSVGPNSPLNDTCAAPDKQQGSVVSAVAKWNKAGIPLEKIVL<br>GVGAYGHAFSVNKTKAYVNGTKELAAYPPFNKDIHPKGDKWDDPAGVDPCGVATPDG<br>GIFTFWGLIEHGYLNEDGSPKRPYRFDNCSRTAYAYNEEEQVMVSFDDAQAFKEKGAFI<br>KSQGLGGFSVWNAGSDHKDILLDAIRSGAGLSK
